# Quantitative lineage tracing strategies to resolve multipotency in tissue-specific stem cells

**DOI:** 10.1101/gad.280057.116

**Published:** 2016-06-01

**Authors:** Aline Wuidart, Marielle Ousset, Steffen Rulands, Benjamin D. Simons, Alexandra Van Keymeulen, Cédric Blanpain

**Affiliations:** 1 Institut de Recherche Interdisciplinaire en Biologie Humaine et Moléculaire (IRIBHM), Université Libre de Bruxelles, Brussels B-1070, Belgium;; 2 Cavendish Laboratory, Department of Physics, University of Cambridge Cambridge CB3 0HE, United Kingdom;; 3 Department of Physiology, Development, and Neuroscience, University of Cambridge, Cambridge CB2 1QN, United Kingdom;; 4 The Wellcome Trust/Cancer Research UK Gurdon Institute, University of Cambridge, Cambridge CB2 1QN, United Kingdom;; 5 Wellcome Trust-Medical Research Council Stem Cell Institute, University of Cambridge, Cambridge CB2 1QR, United Kingdom;; 6 Walloon Excellence in Life Sciences and Biotechnology (WELBIO), Université Libre de Bruxelles, Brussels B-1070, Belgium

**Keywords:** stem cell, progenitor, multipotency, unipotency, mammary gland, prostate

## Abstract

Lineage tracing has become the method of choice to study the fate and dynamics of stem cells (SCs) during development, homeostasis, and regeneration. However, transgenic and knock-in Cre drivers used to perform lineage tracing experiments are often dynamically, temporally, and heterogeneously expressed, leading to the initial labeling of different cell types and thereby complicating their interpretation. Here, we developed two methods: the first one based on statistical analysis of multicolor lineage tracing, allowing the definition of multipotency potential to be achieved with high confidence, and the second one based on lineage tracing at saturation to assess the fate of all SCs within a given lineage and the “flux” of cells between different lineages. Our analysis clearly shows that, whereas the prostate develops from multipotent SCs, only unipotent SCs mediate mammary gland (MG) development and adult tissue remodeling. These methods offer a rigorous framework to assess the lineage relationship and SC fate in different organs and tissues.

## Abstract (precis)

Here, Wuidart et al. present a rigorous new method for assessing the lineage relationship and stem cell fate in different organs and tissues. The authors developed two novel methods for determining lineage relationships: the first one based on statistical analysis of multicolor lineage tracing, and the second one based on lineage tracing at saturation to assess the fate of all stem cells within a given lineage and the “flux” of cells between different lineages.

Tissue-specific stem cells (SCs), which are functionally defined by their ability to self-renew and differentiate, are responsible for tissue morphogenesis during embryonic and postnatal development, during which the different cell types that compose these tissues are specified and expand to generate the cellular diversity and distinct functions of the different adult tissues (Barker et al. 2010; Blanpain and Fuchs 2014). At this stage, tissues stop expanding and enter into a steady-state condition, called tissue homeostasis, in which newly formed cells exactly compensate cell loss. In addition to ensuring the daily replacement of damaged and dead cells, tissue-specific SCs are also activated upon injury and function to repair tissue damage. There are no generic markers of tissue-specific SCs, and these cells are usually described by their functional properties of long-term self-renewal and differentiation (Blanpain and Fuchs 2014). While it was initially assumed that tissue SCs are multipotent, meaning they retain the capacity to differentiate at the single-cell level into different cell lineages, recent studies have demonstrated that unipotent SCs can sustain the development, homeostasis, and repair of many different tissues (Clayton et al. 2007; Wang et al. 2009; Nakagawa et al. 2010; Van Keymeulen et al. 2011; Choi et al. 2012; Lu et al. 2012; Sun et al. 2014).

Historically, the differentiation potential of SCs has been largely studied through the transplantation of purified cells into immuno-deficient mice (Etzrodt et al. 2014). These experiments assess the differentiation potential of the SCs but not necessarily their fate within their natural environment. Under these experimental conditions, it appears that SCs usually present a broader lineage repertoire than under physiological conditions, mimicking what occurs during early morphogenesis or wounding response in adults (Oshima et al. 2001; Morris et al. 2004; Ito et al. 2005; Van Keymeulen et al. 2011; Prater et al. 2014). Lineage tracing allowing the labeling of different cell populations in a spatially and temporally controlled manner in situ has greatly improved our understanding of tissue-specific SC fate within their natural environment in vivo. The most widely used labeling system in mouse models involves the excision of a “floxed” stop cassette upstream of a fluorescent reporter gene by a Cre recombinase expressed under the control of a cell type-specific promoter (Kretzschmar and Watt 2012). The recombinase can be activated upon tamoxifen (TAM) or doxycycline (DOX) administration depending on the system used. This technique allowed us to genetically and irreversibly label cells of interest and follow their fate over time in situ. While this technique has proven to be extremely useful to define the fate of tissue-specific SCs during development, homeostasis, and repair, it can, however, lead to erroneous conclusions when the system is not properly controlled or key parameters are not properly recorded or interpreted, including the lineage specificity of the Cre, the frequency of the labeling (clonal, chimeric, and all cells), and the architecture of the tissues (Kretzschmar and Watt 2012; Blanpain and Simons 2013). While lineage tracing is now used in all fields of developmental and SC biology, no rigorous method has yet been established to interpret with precision and statistical significance the issue of multipotency versus unipotency in lineage tracing experiments.

While clonal analysis, in which a small minority of cells (<1%) is labeled, offers the potential to define multipotency and cell dynamics, such an approach may lead to the preferential labeling of certain subpopulations that are not representative of the whole population of unlabeled cells (Blanpain and Simons 2013). Therefore, most studies use “mosaic” labeling, in which 5%–50% of the cells of a given lineage are labeled. In this case, as in clonal analysis, no information is recovered about the fate of the unlabeled cells. Inducible lineage tracings at saturation, in which all cells of a given lineage are labeled, would provide definitive information about the different cell lineages to which a given SC population can give rise. Unfortunately, this technique has not been used in adult SCs due to the difficulty of labeling all cells within adult tissues following TAM administration. Moreover, a rigorous assessment of the cell lineages initially targeted (i.e., the specificity of the Cre) is critical to properly interpret mosaic lineage tracing data, a key control that tends to be frequently overlooked. These experiments can be particularly misleading if the Cre is not perfectly specific to a given cell lineage but instead targets different cell types within the same tissue. The initial and independent colabeling of adjacent but distinct populations of unipotent progenitors could give the false impression of multipotency.

Here, we developed two analytical methods consisting of (1) statistical analysis of multicolor lineage tracing, allowing the definition of multipotency potential to be achieved with high statistical confidence, and (2) lineage tracing at saturation to assess the fate of all cells within a given lineage and the flux of cells between different lineages during postnatal development and adult remodeling. We performed these experiments in the mammary gland (MG) and the prostate, two ductal epithelial tissues composed of basal cells (BCs) and luminal cells (LCs) for which, despite dozens of independent lineage tracing investigations, the actual fate of their SCs (unipotent, bipotent, or both) remains unclear (Wang et al. 2009, 2013, 2015; Liu et al. 2011; Van Keymeulen et al. 2011; Choi et al. 2012; de Visser et al. 2012; van Amerongen et al. 2012; Lafkas et al. 2013; Lu et al. 2013; Malhotra et al. 2014; Prater et al. 2014; Rios et al. 2014; Tao et al. 2014; Rodilla et al. 2015). Our analysis clearly shows that, while the prostate develops from multipotent SCs, only unipotent SCs mediate MG pubertal expansion and adult tissue remodeling. These methods offer a rigorous framework to assess the lineage relationship and SC fate in different organs and tissues.

## Results

### Colabeling of BCs and LCs by various CreER in the MG

The mouse MG is composed of two cell types: an inner layer of secretory LCs expressing exclusively keratin 8 (K8) and K18 and surrounded by an outer layer of BCs expressing solely basal K5 and K14, also called myoepithelial cells because of their contractile properties (Van Keymeulen et al. 2011). Extensive remodeling and tissue expansion are observed during pubertal development as well as during pregnancy and lactation (Watson and Khaled 2008). Transplantation assays with FACS-isolated Lin^−^CD24^+^CD29^Hi^ BCs regenerate a functional MG, suggesting the existence of multipotent mammary SCs (MaSCs) (Shackleton et al. 2006; Stingl et al. 2006). Lineage tracing experiments using different CreER and DOX-inducible Cre showed that both basal (K5/K14) and luminal (K8/K18) populations are maintained by unipotent basal and luminal SCs in the postnatal MG as well as during pregnancy. Different lineage tracing studies demonstrated that unipotent myoepithelial cells under normal physiological conditions are multipotent in transplantation assays (Van Keymeulen et al. 2011; Prater et al. 2014). Other studies tracing either BCs (Lgr5, Axin2, and Ad-K14-Cre) or LCs (Notch1, Notch3, Lgr5, Axin2, and Ad-K8-Cre) further supported the notion that both lineages in the MG are self-sustained by unipotent SCs in vivo during MG postnatal development and homeostasis and adult remodeling (Van Keymeulen et al. 2011; de Visser et al. 2012; van Amerongen et al. 2012; Lafkas et al. 2013; Prater et al. 2014; Tao et al. 2014; Rodilla et al. 2015). However, two recent studies using new K14 and K5 transgenic and Procr-CreER knock-in mice suggested instead that BCs contain multipotent SCs (Rios et al. 2014; Wang et al. 2015). Thus, it remains unclear what the cause of the major discrepancy is between these different studies, raising the question of whether multipotent SCs do indeed exist and, if so, what their relative contribution to MG development is and their frequency as compared with unipotent SCs.

**Figure 1.**
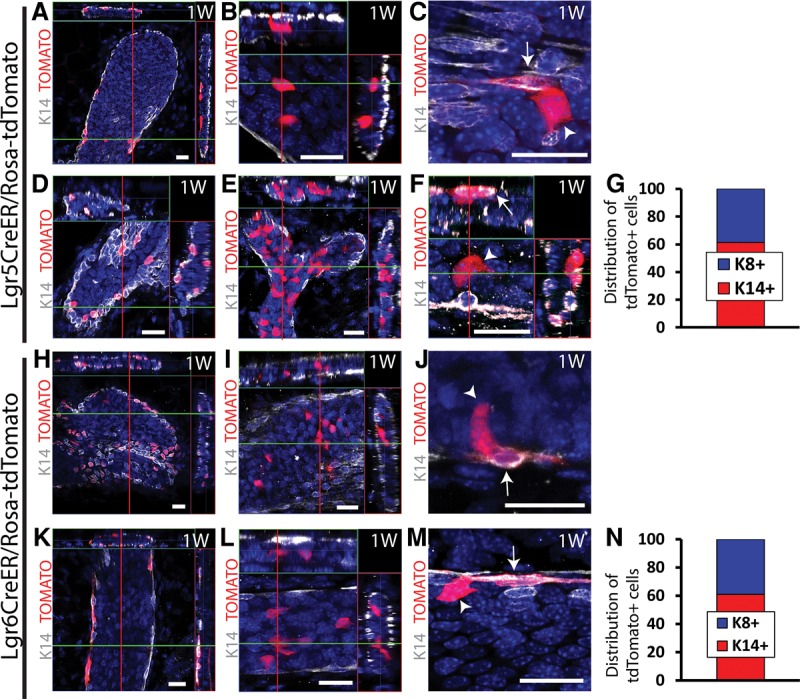
Colabeling of BCs and LCs by Lgr5 and Lgr6CreER in the MG. (*A*–*F*) Confocal imaging of immunostaining of K14 and Tomato 1 wk after TAM administration to Lgr5-EGFP-IRES-CreER^T2^/Rosa-tdTomato pubertal (15 mg) (*A*–*C*) or adult virgin (1.5 mg) (*D*,*E*) mice. (*A*,*D*) Isolated BCs. (*B*,*E*) Isolated LCs. (*C*,*F*) Doublets of BCs and LCs. In adult mice, doublets could be observed only at high doses of TAM (15 mg). (*G*) Distribution of basal K14^+^ and luminal K8^+^ cells among Tomato^+^ cells (2865 cells out of three mice). (*H–M*) Confocal imaging of immunostaining of K14 and Tomato cells 1 wk after TAM administration (15 mg) to Lgr6-EGFP-IRES-CreER^T2^/Rosa-tdTomato pubertal (*H*–*J*) or adult virgin (*K*–*M*) mice. (*H*,*K*) Isolated BCs. (*I*,*L*) Isolated LCs. (*J*,*M*) Doublets of BCs and LCs. (*N*) Distribution of basal K14^+^ and luminal K8^+^ cells among Tomato^+^ cells (4683 cells out of three mice). Arrows depict BCs, and arrowheads depict LCs. (*A*,*B*,*D*–*F*,*H*,*I*,*K*,*L*) Orthogonal projections of three-dimensional (3D) stacks. (*C*,*J*,*M*) Single-plane images from a 3D stack. Bars, 20 µm. Histograms represent the mean. See Supplemental Table S1 for further information on the statistics.

To define the cellular heterogeneity of BCs and LCs in the MG, we performed lineage tracing with different knock-in and transgenic CreER mice that have been shown to mark epithelial SCs in different organs and tissues and analyzed the fate of targeted cells during pubertal development and in adult mice (Barker et al. 2007; Means et al. 2008; Nowak et al. 2008; Snippert et al. 2010a; Malhotra et al. 2014). In the absence of TAM administration, no labeled cells were observed in any of these CreER mice during puberty or adulthood, demonstrating the absence of leakiness of these mice (data not shown). Confocal analysis of whole mounts (WMs) of MGs 1 wk following TAM administration to Lgr5CreER^T2^/Rosa-tdTomato and Lgr6CreER^T2^/Rosa-tdTomato 4- or 8-wk-old mice showed that these CreER preferentially targeted BCs (Fig. 1A,D,H,K) (61.3% and 61% of Tomato^+^ cells were BCs in Lgr5 [Fig. 1G; Supplemental Table S1] and Lgr6 [Fig. 1N; Supplemental Table S1] mice, respectively), although a fraction of LCs was also labeled (Fig. 1B,E,I,L). The presence of labeled LCs was similar in Lgr5 and Lgr6 mice (38.7% and 39%, respectively) (Fig. 1G,N; Supplemental Table S1). These labeled LCs were induced initially and independently of BCs, as shown by the 11% of LCs as early as 48 h after TAM administration (Supplemental Fig. S1A–C; Supplemental Table S7). Labeled BCs and LCs were found mostly as isolated labeled cells, defined as labeled BCs or LCs that do not touch any labeled LCs or BCs (Fig. 1A,B,D,E,H,I,K,L; Supplemental Fig. S1A,B), demonstrating that these CreER are not completely specific to BCs and can also initially target LCs. Groups of contiguous labeled cells touching each other (termed “doublets” here) containing BCs and LCs were observed in both Lgr5 and Lgr6 only following higher doses of TAM that lead to a nonlinear increase in the chimerism (Fig. 1C,F,J,M; Supplemental Fig. S1D), potentially suggesting that the BC lineage may contain a rare subpopulation of bipotent SCs that is only labeled at higher doses of TAM (Supplemental Fig. S1D; Supplemental Table S7). However, the absence of doublets observed when a lower dose of TAM was administered suggests that these doublets could be explained by the random initial labeling of two independent but neighboring BCs and LCs.

**Figure 2.**
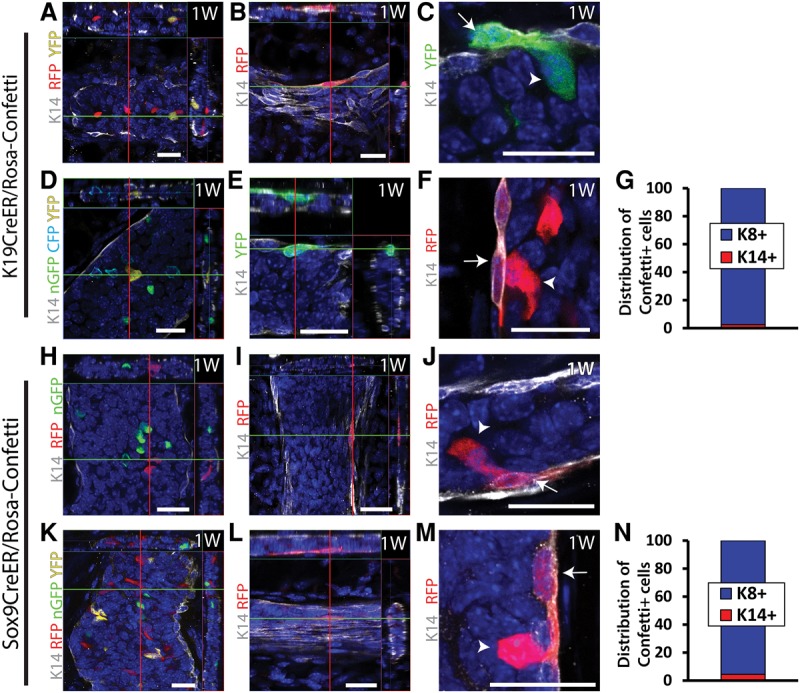
Colabeling of LCs and BCs by K19 and Sox9CreER in the MG. (*A*–*F*) Confocal imaging of immunostaining of K14 and Confetti cells 1 wk after TAM administration (15 mg) to K19CreER^T^/Rosa-Confetti pubertal (*A*–*C*) or adult virgin (*D*–*F*) mice. (*A*,*D*) Isolated LC. (*B*,*E*) Isolated BCs. (*C*,*F*) Doublets of BCs and LCs. (*G*) Distribution of basal K14^+^ and luminal K8^+^ cells among Confetti^+^ cells (21,408 cells out of three mice). (*H*–*M*) Confocal imaging of immunostaining of K14 and Confetti cells 1 week after TAM administration (5 mg) to Sox9CreER^T2^/Rosa-Confetti pubertal (*H*–*J*) or adult virgin (*K*–*M*) mice. (*H*,*K*) Isolated LCs. (*I*,*L*) Isolated BCs. (*J*,*M*) Doublets of BCs and LCs. (*N*) Distribution of basal K14^+^ and luminal K8^+^ cells among Confetti^+^ cells (4258 cells out of three mice). Arrows depict BCs, and arrowheads depict LCs. (*A*,*B*,*D*,*E*,*H*,*I*,*K*,*L*) Orthogonal projections of 3D stacks. (*C*,*F*,*J*,*M*) Single-plane images from a 3D stack. Bars, 20 µm. Histograms represent the mean. See Supplemental Table S2 for further information on the statistics.

Lineage tracing using knock-in K19CreER^T^/Rosa-Confetti and transgenic Sox9CreER^T2^/Rosa-Confetti mice during puberty or homeostasis leads to the labeling of mostly LCs (Fig. 2A,D,H,K) (97.4% and 95.6% of the labeled cells were LCs in K19CreER^T^ [Fig. 2G; Supplemental Table S2] and Sox9CreER^T2^ [Fig. 2N; Supplemental Table S2] adult mice, respectively). A small fraction of BCs (2.6% and 4.4%, respectively) (Fig. 2G,N) was also labeled using these CreER (Fig. 2B,E,I,L). At this mosaic level of cell labeling, the vast majority of labeled cells was found as isolated labeled cells (Fig. 2A,B,D,E,H,I,K,L), but some rare doublets of BCs and LCs could also be found (Fig. 2C,F,J,M), potentially suggesting that some rare LCs could also be bipotent, giving rise to BCs.

Our mosaic lineage tracing experiments in the MG showed that many knock-in and transgenic CreER mice commonly used to mark epithelial SCs in different tissues, including Lgr5, Lgr6, K19, and Sox9, lead to the initial and independent labeling of both BCs and LCs, as demonstrated by the presence of isolated labeled cells of both lineages. This lack of specificity for a given lineage during development and homeostasis complicates the interpretation of the doublets of BCs and LCs labeled with the same fluorescent color, which could arise from either the initial and independent labeling of two adjacent unipotent cells or the differentiation of a bipotent SC.

### Biostatistical analysis of mosaic multicolor tracing demonstrates the unipotency of basal MG SCs during development and adult regeneration

**Figure 3.**
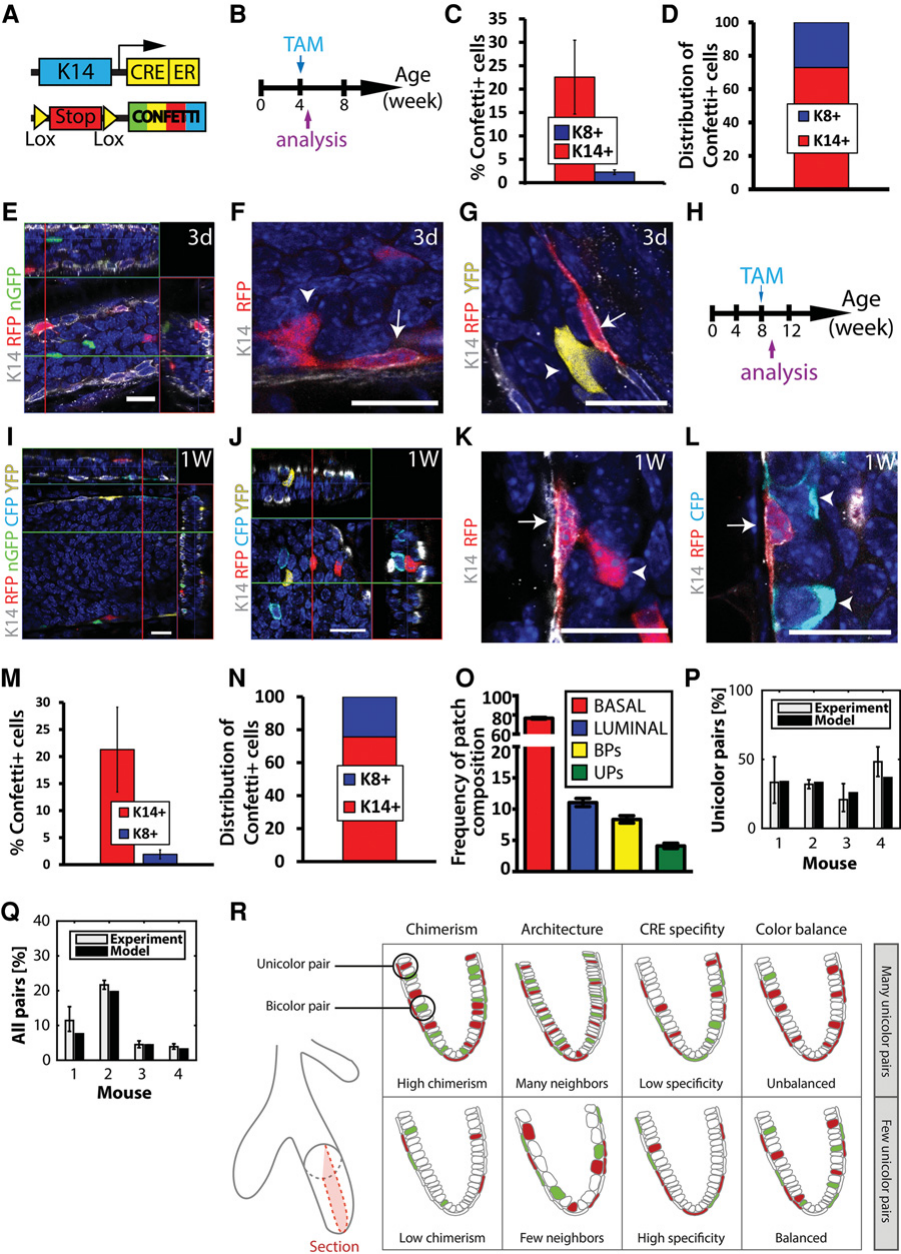
K14CreER^T2^ targets initially and independently unipotent BCs and LCs in the MG. (*A*) Scheme summarizing the genetic strategy used to target Confetti expression in K14-expressing cells. (*B*) Scheme summarizing the protocol used to study the fate of cells targeted at puberty using K14CreER^T2^/Rosa-Confetti mice. (*C*,*D*) Percentage of Confetti^+^ cells in basal K14^+^ and luminal K8^+^ cells (*C*) and distribution of basal K14^+^ and luminal K8^+^ cells among Confetti^+^ cells (*D*) 3 d after TAM injection (1.5 mg) at puberty in K14CreER^T2^/Rosa-Confetti mice (15524 cells out of three mice). (*E*–*G*) Confocal imaging of immunostaining of K14 and Confetti 3 d after TAM administration (1.5 mg) at puberty in K14CreER^T2^/Rosa-Confetti mice. Examples of independently and initially labeled BCs and LCs (*E*), UPs (*F*), and BPs (*G*) containing BCs and LCs. (*H*) Scheme summarizing the protocol used to study the fate of cells targeted in adulthood using K14CreER^T2^/Rosa-Confetti mice. (*I*–*L*) Confocal imaging of immunostaining of K14 and Confetti at 1 wk after TAM administration (1.5 mg) to K14CreER^T2^/Rosa-Confetti adult virgin mice. Examples of independently and initially labeled BCs (*I*) and LCs (*J*) as well as UPs (*K*) and BPs (*L*) containing BCs and LCs. (*M*,*N*) Percentage of Confetti^+^ cells in basal K14^+^ and luminal K8^+^ cells (*M*) and distribution of K14^+^ BCs and K8^+^ LCs among Confetti^+^ cells (*N*) 1 wk after TAM injection (1.5 mg) to K14CreER^T2^/Rosa-Confetti adult virgin mice (55287 cells out of five mice). (*O*) Frequency of Confetti^+^ patch compositions 1 wk after TAM injection (1.5 mg) to K14CreER^T2^/Rosa-Confetti adult virgin mice. (*P*) Fraction of UPs among all pairs 1 wk after TAM injection (1.5 mg) to K14CreER^T2^/Rosa-Confetti adult virgin mice. Experimental bars (gray) represent the number of UPs divided by the total number of pairs. Model bars (black) represent the prediction of the fraction of UPs among all pairs, which depends only on the relative frequencies of the Confetti colors. (*Q*) Fraction of total pairs 1 wk after TAM injection (1.5 mg) to K14CreER^T2^/Rosa-Confetti adult virgin mice. Experimental bars (gray) represent the total number of pairs divided by the total number of labeled BCs. Model bars (black) represent the prediction of the overall fraction of pairs, which is based on the probability of random labeling of BCs. Although a formal expression is derived in the Supplemental Material, this fraction is roughly proportional to three factors: the degree of chimerism, the specificity of the CRE, and the architecture of the tissue (i.e., how many LCs touch a BC). (*R*) Scheme summarizing the parameters taken into account in the model. In a hypothetical situation with only two colors, variation of any of these parameters influences the observed number of UPs. Arrows depict BCs, and arrowheads depict LCs. (*E*,*I*,*J*) Orthogonal projections of 3D stack. (*F*,*G*,*K*,*L*) Single-plane images from a 3D stack. Bars, 20 µm. Error bars represent mean ± SEM, except in *O*, where it represents one-σ binomial confidence levels for the observed frequencies of patch compositions, and in *P* and *Q*, where it represents standard deviation. See Supplemental Table S3 for further information on the statistics.

To overcome the difficulty of interpreting such lineage tracing data and define whether two adjacent cells labeled with the same reporter represent a multipotent SC or the random labeling of two neighboring unipotent cells, we developed a novel biostatistical framework to infer with high confidence the existence of multipotent and/or unipotent SCs in different tissues. To this end, we used the same K14CreER^T2^/Rosa-Confetti mouse model that was recently reported to target bipotent basal MaSCs (Rios et al. 2014). Female mice were induced with 1.5 mg of TAM at puberty (4 wk) or in adulthood (8 wk), and three-dimensional (3D) WMs of the MG were analyzed by confocal microscopy 3 d or 1 wk following TAM administration, respectively (Fig. 3A,B,H). With this dose of TAM, ∼20% of BCs were labeled (Fig. 3C,E,I,M). Among the labeled cells, the majority were BCs (Fig. 3D,N; Supplemental Table S3). However, this K14CreER^T2^ also induced the labeling of ∼2% of LCs (Fig. 3C,E,J,M), similar to the proportion of labeled LCs 2 d after DOX administration to K5rtTA/TetOCre/Rosa-Confetti mice (Rios et al. 2014). Because of the higher number of LCs over BCs, these 2% of labeled cells within all LCs represent ∼24% of all labeled cells 1 wk following TAM administration during both pubertal development and adult homeostasis (Fig. 3D,N; Supplemental Table S3). In the majority of the cases, the labeled cells consisted of either isolated BCs or LCs (single cells or groups of cells) not in contact with any other marked cell (Fig. 3E,I,J,O), demonstrating the lack of specificity of this CreER for the BCs. Doublets of BCs and LCs (defined as touching each other) in which both cells were labeled with either the same color (referred to here as unicolor pair [UP]) or two different colors (referred to here as bicolor pair [BP]) were also observed (Fig. 3F,G,K,L). Quantification of the composition of each patch of cells labeled with the different Confetti reporter proteins and touching each other in adult mice revealed that 77% of the unicolor patches corresponded to isolated BCs, 11% corresponded to isolated LCs, and 4% corresponded to UPs, while 8% BPs were observed (Fig. 3O; Supplemental Table S3).

We next asked whether the observed frequencies of UPs reflect the clonal expansion of induced bipotent BCs or can be explained by chance due to the independent labeling of two neighboring BCs and LCs. To this end, we developed a rigorous biostatistical method to address the mosaic data. This mathematical analysis comprises two steps: First, we assessed the probability that any given pair is unicolor. Second, we assessed the probability that any given labeled BC comes in contact with an independently labeled LC. In other words, we sought a rigorous assessment of how UPs are generated.

We began by analyzing the fraction of UPs among all pairs, given by the observed number of UPs divided by the total number of UPs and BPs (Fig. 3P, gray bar on the graph). This fraction was then compared with what one would expect theoretically if UPs derived exclusively from the chance of labeling neighboring unipotent BCs and LCs (Fig. 3P, black bar on the graph). To determine the latter, we first introduced the parameters $r_{B}^{C}$ and $r_{L}^{C}$, defined as the relative frequencies of contiguous labeled patches of a given Confetti color, *C*, in the basal and luminal layers, respectively, given by the number of each Confetti color^+^ cells (RFP^+^, YFP^+^, nGFP^+^, or mCFP^+^ cells) divided by the total number of Confetti^+^ cells. If BCs and LCs are unipotent, then the expected fraction of all pairs that are unicolor is simply given by the probability that a given pair is labeled in the same color, $\sum_{C}r_{L}^{C}r_{B}^{C}$ (Fig. 3P; see the “Statistical Analysis” section for further details). With this definition, any observed excess of UPs over that predicted by chance labeling of neighboring BCs and LCs would provide evidence for bipotency. However, comparison of the experimental fraction with the theoretical prediction (Fig. 3P) shows that the measured frequency of UPs is entirely consistent with the unipotency of BCs and LCs (*P* = 0.65). We therefore concluded that, on the basis of the statistical analysis of the Confetti labeling data, there is no evidence in support of bipotency. However, by itself, this analysis does not allow us to rule out the potential for a minority contribution of bipotent cells to MG development.

To further challenge our conclusion of unipotency and assess the predictive value of the chance labeling hypothesis, in the second step of our analysis, we calculated the fraction of labeled BCs that are paired by proximity with a labeled LC. The latter is given simply by the observed total number of pairs divided by the total number of labeled BCs (Fig. 3Q, gray bar). Once again, this fraction can be compared with the theoretical prediction obtained from considering the chance of labeling unipotent BCs and LCs. To perform this comparison, one must take into account the cellular architecture of the tissue or coordination number (i.e., how many LCs, on average, are in physical contact with a BC and therefore are considered neighbors), the degree of chimerism (i.e., the relative fraction of labeled BCs and LCs among all epithelial cells), the specificity of the Cre (i.e., the relative frequency of labeled BCs or LCs), and the relative frequency of recombination events associated with each Confetti color as defined above (Fig. 3R; see the “Statistical Analysis” section for further details). With these parameters defined, we started by determining the probability that a marked BC of color C1 lies in proximity to a marked LC of color C2, a calculation that depends on the number of luminal neighbors of this cell. Next, taking into account the relative induction frequencies of the different colors and the fact that the coordination between BCs and LCs is variable (ranging from three to seven LCs for one BC) (see Table 3 in the “Statistical Analysis” section), we obtained an expression for the expected fraction of paired labeled BC patches (σ), which depends nontrivially on the degree of chimerism (*p*), the specificity of the Cre (*s*), and the coordination number ($\bar{z}$). In particular, σ depends nontrivially on the luminal induction frequency: The chance of labeled pairs increases with the induction frequency. By taking into account all statistical permutations, a detailed expression for the fraction, σ, can be derived (see the “Statistical Analysis” section). However, if the degree of chimerism is almost clonal and coordination is invariant, the fraction of paired BCs assumes the simple dependence $\sigma \approx \;\lambda \;\bar{z}$, where the luminal induction probability (λ) is related to the overall degree of chimerism, the Cre specificity, and the relative frequency of LCs among all cells (*f*) through the relation λ = *p* × *f*/*s*. Once again, comparison of the predicted fraction with the experimental data (Fig. 3Q) shows that the data are consistent with a model in which BCs and LCs function as unipotent cells.

In conclusion, we found that our simple model can accurately predict both the frequency of UPs and the total frequency of pairs in the MG and provided strong evidence that BCs in the pubertal and adult MG are unipotent.

To directly assess whether the labeled LCs arise from independent labeling of LCs and not from the division of a basal multipotent cells, we treated K14CreER^T2^/Rosa-Confetti with 1.5 mg of TAM together with continuous administration of BrdU during the 3 d of chase (Supplemental Fig. S2A). Confocal analysis of WM MGs showed that 75% of labeled BCs and 78% of labeled LCs were BrdU-negative after 3 d of chase, demonstrating that most of the labeled LCs did not divide during the chase period (Supplemental Fig. S2B,C; Supplemental Table S8). In addition, the remaining 22% of labeled LCs that were BrdU^+^ were not associated with a labeled BC (Supplemental Fig. S2B,D), further demonstrating that the labeled LCs are marked independently from the BCs by this K14CreER^T2^ mouse and did not arise from the division of multipotent basal SCs.

Interestingly, when the dose of TAM was drastically reduced to perform clonal analysis with the same mice, only isolated BCs and LCs were targeted during puberty (0.05 mg of TAM) (Supplemental Table S9; Supplemental Fig. S3A–D) and adult homeostasis (0.25 mg of TAM) (Supplemental Table S9; Supplemental Fig. S3G–J). Clonal expansion of BCs (Supplemental Fig. S3E,K) or LCs (Supplemental Fig. S3F,L) 4 wk later suggested that K14CreER initially targeted unipotent BCs and LCs. Moreover, no doublets were observed following a clonal dose of TAM, showing that the appearance of doublets occurs at a higher level of chimerism. Altogether these data support the notion that these UPs arise by chance from the independent and initial labeling of BCs and LCs and do not support the existence of bipotent MG SCs during pubertal development and adult remodeling.

### Biostatistical analysis of multicolor clonal tracing demonstrates the multipotency of basal prostate SCs during development

The adult prostate is a pseudostratified epithelium composed of BCs and LCs expressing K5 and K14 or K8 and K18, respectively (Shen and Abate-Shen 2010). Lineage tracing experiments during prostate postnatal development, adult homeostasis, and adult regeneration after castration suggested that prostate postnatal development is mediated by multipotent and unipotent SCs, whereas adult prostate maintenance and regeneration are mediated by basal and luminal unipotent SCs (Wang et al. 2009, 2013, 2014; Liu et al. 2011; Choi et al. 2012; Ousset et al. 2012; Lu et al. 2013; Pignon et al. 2013).

**Figure 4.**
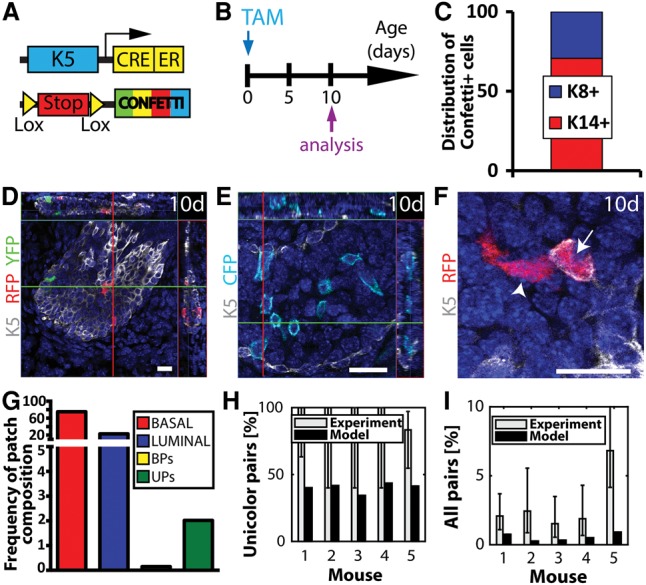
K5CreER^T2^ targets multipotent BCs that give rise to LCs in the prostate. (*A*) Scheme summarizing the genetic strategy used to induce Confetti expression in K5-expressing cells during prostate postnatal development. (*B*) Scheme summarizing the protocol used to study the fate of cells targeted during postnatal development using K5CreER^T2^/Rosa-Confetti mice. (*C*) Distribution of basal K14^+^ and luminal K8^+^ cells among Confetti^+^ cells 10 d after TAM (0.1 mg) administration at birth to K5CreER^T2^/Rosa-Confetti mice (1268 cells out of five mice). (*D*–*F*) Confocal imaging of immunostaining of K5 and fluorescent Confetti cells 10 d after TAM (0.1 mg) administration at birth to K5CreER^T2^/Rosa-Confetti mice. Isolated BCs (*D*) and LCs (*E*) as well as UPs containing BCs and LCs (*F*) were observed. In *D*, the section depicted represents the surface of the duct, meaning that the cells expressing K5 are located at the surface of the duct and not inside. This is illustrated by the red and green lines in the orthogonal sections of the WM. (*G*) Frequency of Confetti^+^ patch compositions 10 d after TAM (0.1 mg) injection at birth to K5CreER^T2^/Rosa-Confetti mice. (*H*) Fraction of UPs among all pairs 10 d after TAM (0.1 mg) administration at birth to K5CreER^T2^/Rosa-Confetti mice. Experimental bars (gray) represent the number of UPs divided by the total number of pairs. Model bars (black) represent the prediction of the fraction of UPs among all pairs, which depends only on the relative frequencies of the Confetti colors. (*I*) Fraction of total pairs 10 d after TAM (0.1 mg) administration at birth to K5CreER^T2^/Rosa-Confetti mice. Experimental bars (gray) represent the total number of pairs divided by the total number of induced BCs (approximated by the number of cohesive groups of BCs). Model bars (black) represent the prediction of the overall frequencies of pairs, which is based on the probability of random labeling of BCs, taking into account the architecture of the tissue (i.e., how many LCs touch a BC), the degree of chimerism, the CRE specificity, and the frequency of recombination of each Confetti color. (*D*,*E*) Orthogonal projections of 3D stacks. (*F*) Single-plane image from a 3D stack. Bars, 20 µm. Histograms represent the mean. Error bars represent standard deviation. See Supplemental Table S4 for further information on the statistics.

To challenge our biostatistical analysis of multipotency, we performed multicolor mosaic tracing during prostate postnatal development, during which multipotent SCs have been suggested to contribute to morphogenesis (Ousset et al. 2012; Pignon et al. 2013; Wang et al. 2014). K5CreER^T2^/Rosa-Confetti newborn mice (postnatal day 1 [P1]) were induced with a single low dose of TAM (0.1 mg) and analyzed 10 d later (Fig. 4A,B). Quantification of confocal imaging of microdissected WMs of the prostate showed that, while the vast majority of recombined cells was isolated (single or groups of) BCs (Fig. 4C,D) or LCs not touching any other labeled cell (Fig. 4C,E; Supplemental Table S4), we could readily observe a small fraction of UPs (Fig. 4F,G). Interestingly, with this dose of TAM, almost no BPs were observed (0.15% of clones, which corresponds to one BP out of 775 counted patches) (Fig. 4G; Supplemental Table S4), highlighting the strong enrichment of UPs compared with BPs.

To assess the potential bipotency of the progenitor pool, we performed the same statistical analysis of the data set, considering the same series of parameters as the one used for the MG and adjusted for the prostate (i.e., the degree of chimerism, the Cre specificity, the relative frequencies of recombination of Confetti colors, and the specific architecture of prostate tissue). Despite a clonal induction frequency, we observed frequent unicolor groups of cells in the basal and luminal layers, indicating a significant amount of cell divisions between labeling and analysis. As these cohesive patches are clonal with high probability, we considered touching groups of (unicolor) cohesive patches in the basal and luminal layer as (unicolor) pairs. In contrast to the behavior of the MG, we found that both the frequency of UPs among all pairs (Fig. 4H) and the total frequency of patches (Fig. 4I) were consistently higher than what one could expect from merely the chance labeling of neighboring BCs and LCs. Rather, the excess of UPs is highly likely to arise from the local expansion of marked multipotent BCs that fuel prostate postnatal development (*P* = 0.0064) (Fig. 4H; “Statistical Analysis” section). These results demonstrate the power of statistical analysis to resolve with high confidence the question of SC multipotency during postnatal development and adult homeostasis.

### Lineage tracing at saturation demonstrates that all basal MG SCs are unipotent during development and adult regeneration

**Figure 5.**
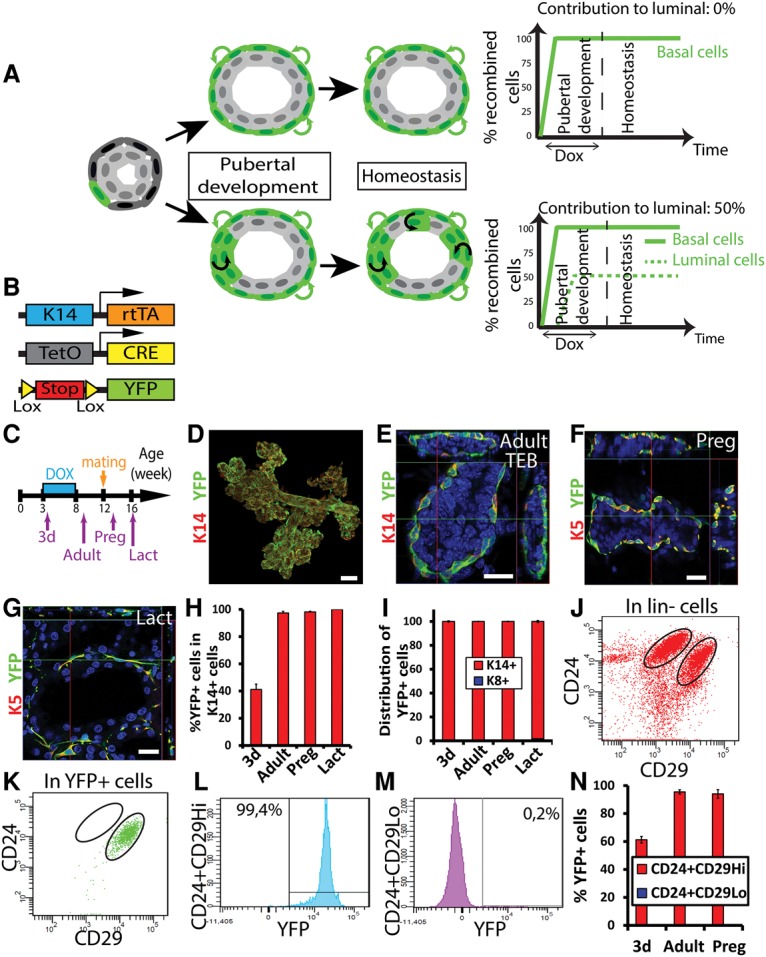
BCs are a self-sustained unipotent lineage in the MG during puberty and adult remodeling. (*A*) Theoretical outcomes of tracing BCs at saturation: BCs can be either a self-sustained unipotent lineage (*top* panel) or a lineage comprising bipotent SCs that produce labeled LCs over time (*bottom* panel). (*B*) Scheme summarizing the genetic strategy used to target YFP expression in K14rtTA/TetO-Cre/Rosa-YFP mice. (*C*) Scheme summarizing the protocol used to study the fate of cells targeted during puberty using K14rtTA/TetO-Cre/Rosa-YFP mice. (*D*–*G*) Confocal imaging of immunostaining of K14 or K5 and YFP at low magnification (*D*), at the adult stage (*E*), during pregnancy (*F*), and during lactation (*G*) in K14rtTA/TetO-Cre/Rosa-YFP mice treated for 5 wk with DOX at puberty. (*H*,*I*) Percentage of YFP^+^ cells in basal K14^+^ (*H*) and distribution of basal K14^+^ and luminal K8^+^ cells among YFP^+^ cells (*I*) 3 d after induction, in adult virgin mice, during pregnancy, and during lactation in K14rtTA/TetO-Cre/Rosa-YFP mice treated for 5 wk with DOX at puberty (12,945, 11,346, 19,609, and 25,583 cells out of three mice per time point). (*J*–*M*) FACS analysis of CD24 and CD29 expression in Lin^−^ (*J*) and Lin^−^YFP^+^ (*K*–*M*) cells in adult virgin K14rtTA/TetO-Cre/Rosa-YFP mice treated for 5 wk with DOX at puberty. (*N*) Percentage of YFP^+^ cells in Lin^−^CD24^+^CD29^Hi^ and Lin^−^CD24^+^CD29^Lo^ populations 3 d after induction, at the adult stage, and during pregnancy in K14rtTA/TetO-Cre/Rosa-YFP mice treated for 5 wk with DOX at puberty. (*D*–*G*) Orthogonal projections of 3D stacks. Bars: *D*, 50 µm; all others, 20 µm. Error bars represent mean ± SEM. See Supplemental Table S5 for further information on the statistics.

Rare bipotent SCs could escape the labeling at clonal or mosaic density because they do not express the gene targeted by the promoter. To avoid this caveat, one needs to label all of the cells of a given cell lineage. Classical lineage tracing experiments using a CreER are often limited in terms of levels of recombination due to TAM toxicity at high doses. In order to circumvent TAM toxicity and achieve the highest level of recombination possible, we used DOX-inducible (Tet-On) mice to perform lineage tracing at saturation, relying on a long-term administration of DOX and allowing reporter recombination at very high chimerism, very close to labeling every single cell of a given lineage (95%–99% of labeled cells) without any toxicity or impairment of MG development. Using such lineage tracing at saturation, it is possible in theory to precisely examine the proportion of putative cells that are bipotent and transit from the BCs to the LCs within the whole MG during postnatal development and throughout the life of the animals (Fig. 5A). For example, if no LCs are initially labeled and then all LCs eventually become labeled, this means that only bipotent SCs contribute to the luminal lineage. If 50% of LCs are eventually labeled, this means that 50% of the LCs arise from bipotent basal SCs (Fig. 5A, bottom panel). If no LCs become labeled, this rules out the existence of bipotent cells within the basal lineage if all BCs are labeled (Fig. 5A, top panel).

To this end, DOX was administered continuously to K14rtTA/TetO-Cre/Rosa-YFP mice during the whole course of pubertal development, and the fate of labeled cells was assessed at different time points (after 3 d of DOX, in adult mice, during pregnancy, and during lactation) by confocal microscopy and FACS analysis of the whole MG (Fig. 5B,C). Importantly, although mice were labeled during puberty to achieve the complete recombination of BCs at the end of pubertal development, these experiments were designed to assess the fate of all BCs in adult mice.

Quantification following 3D confocal analysis of the WM MG demonstrated that, 6 wk after the initiation of the DOX administration, almost all BCs (97%) were YFP-labeled, and only 0.04% of LCs were YFP^+^ (Fig. 5D,E,H,I; Supplemental Table S5) despite the analysis of >10,000 cells per mouse in three different mice per time point, showing that, during postnatal development, all BCs expressing K5/K14 are unipotent and ensure myoepithelial lineage expansion solely and do not contribute to the generation of LCs. Analysis of labeled cells during pregnancy and lactation revealed that, despite the complete labeling of the myoepithelial cells (98%–100%), no YFP^+^-labeled cells were observed in the luminal lineage (0.04%–0.2%) during adult tissue remodeling (Fig. 5F–I). As shown in Figure 5I and quantified in three different mice per time point, almost all YFP^+^ cells were BCs in adult virgin mice (99.8%), during pregnancy (99.85%), and during lactation (98.3%) (Fig. 5I; Supplemental Table S5).

To quantify in a precise and unbiased manner several million epithelial cells corresponding to the whole MG, we performed FACS quantification using CD29/CD24 markers, which have been demonstrated to readily distinguish BCs (CD24^+^CD29^Hi^) from LCs (CD24^+^CD29^Lo^) (Shackleton et al. 2006; Stingl et al. 2006). Analysis of YFP expression in CD24^+^CD29^Hi^ cells showed that up to 99% of the BCs were labeled (average, 95.5%) (Fig. 5J–L), while only 0.2% of the LCs (CD24^+^CD29^Lo^) were YFP-positive (Fig. 5M). This very small proportion of LCs was observed as soon as 3 d following DOX administration (Fig. 5N) and did not increase over time (Fig. 5N), demonstrating the limit of the specificity in the BC labeling using the K14rtTA driver (0.2% of luminal contamination), which is by far the most specific and precise system to induce complete genetic recombination in the BC lineage of the MG.

These results highlight the extremely high efficiency and specificity of labeling that is reached with this system. Together, these results rule out any significant contribution of K14^+^ bipotent SCs to the luminal lineage and show that the immense majority of, if not all, K14^+^ BCs are maintained by unipotent SCs under physiological conditions during MG pubertal expansion, pregnancy, and lactation.

### Lineage tracing at saturation demonstrates the long-term renewal potential of luminal SCs and the fact that LCs are not replaced by basal SCs over time

**Figure 6.**
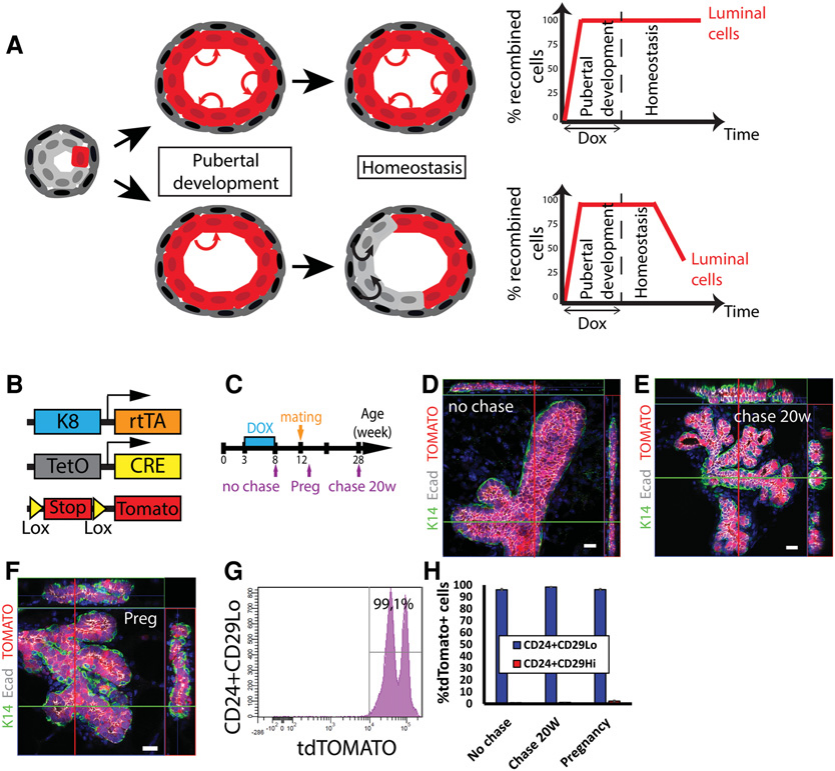
LCs are a self-sustained unipotent lineage in the MG during puberty and adult remodeling. (*A*) Theoretical outcomes of tracing LCs at saturation: LCs can be either a self-sustained unipotent lineage (*top* panel) or a lineage comprising luminal progenitors that are replaced over time by bipotent basal SCs (*bottom* panel). (*B*) Scheme summarizing the genetic strategy used to target Tomato expression in K8rtTA/TetO-Cre/Rosa-tdTomato mice. (*C*) Scheme summarizing the protocol used to study the fate of cells targeted during puberty using K8rtTA/TetO-Cre/Rosa-tdTomato mice. (*D*–*F*) Confocal imaging of immunostaining of K14 and E-cadherin and fluorescent Tomato cells at the end of treatment (*D*), at 20 wk after induction (*E*), and during pregnancy (*F*) in K8rtTA/TetO-Cre/Rosa-tdTomato mice pulsed for 5 wk with DOX at puberty. (*G*) FACS analysis of CD24 and CD29 expression in Lin^−^Tomato^+^ cells at the end of treatment in K8rtTA/TetO-Cre/Rosa-tdTomato mice treated for 5 wk with DOX at puberty. (*H*) Percentage of Tomato^+^ cells in Lin^−^CD24^+^CD29^Hi^ and Lin^−^CD24^+^CD29^Lo^ populations at the end of treatment, at 20 wk after induction, and during pregnancy in K8rtTA/TetO-Cre/Rosa-tdTomato mice pulsed for 5 wk with DOX at puberty. (*D*–*F*) Orthogonal projections of 3D stacks. Bars, 20 µm. Error bars represent mean ± SEM.

Conflicting results have been obtained regarding whether the luminal lineage contains a population of long-term unipotent SCs (Fig. 6A, top panel) or whether the luminal lineage consists of committed progenitors with short-term self-renewal potential that are replaced by basal SCs (Fig. 6A, bottom panel; Van Keymeulen et al. 2011; de Visser et al. 2012; van Amerongen et al. 2012; Lafkas et al. 2013; Malhotra et al. 2014; Rios et al. 2014; Tao et al. 2014; Rodilla et al. 2015).

We performed luminal lineage tracing at saturation to determine the long-term renewal potential of the LC population and whether a significant fraction of labeled LCs is progressively replaced by another unlabeled SC population (Fig. 6A, bottom panel) that is not targeted using our K14 lineage tracing experiments. To this end, we administered DOX during pubertal development to DOX-inducible K8rtTA/TetO-Cre/Rosa-tdTomato mice (Fig. 6B). Expression of tdTomato in LCs was assessed over time in adult and pregnant mice (Fig. 6C).

At the end of the DOX treatment, confocal microscopy of WMs of MGs showed the complete labeling of LCs and the absence of BCs labeled in this condition (Fig. 6D). If the luminal lineage was composed of a population of relatively short-lived progenitors, the percentage of labeled cells would be expected to drop dramatically over time (Fig. 6A, bottom panel). However, 5 mo after DOX administration, the proportion of labeled LCs remained constant, as they were not replaced by unlabeled cells (Fig. 6E). Likewise, no decrease in the proportion of labeled LCs was observed during pregnancy (Fig. 6F). FACS analysis confirmed that >98% of the LCs (CD24^+^CD29^Lo^) were Tomato^+^ during adult homeostasis, during pregnancy, and after 5 mo of chase (Fig. 6G,H), demonstrating that the luminal lineage is self-sustained and maintained by a population of unipotent SCs that present long-term self-renewal potential in adult mice during the estrus cycle and pregnancy and are not replaced over time by putative bipotent SCs (Fig. 6A, top panel).

### Lineage tracing at saturation demonstrates the multipotency of basal prostate SCs and their flux toward the luminal lineage during prostate development

**Figure 7.**
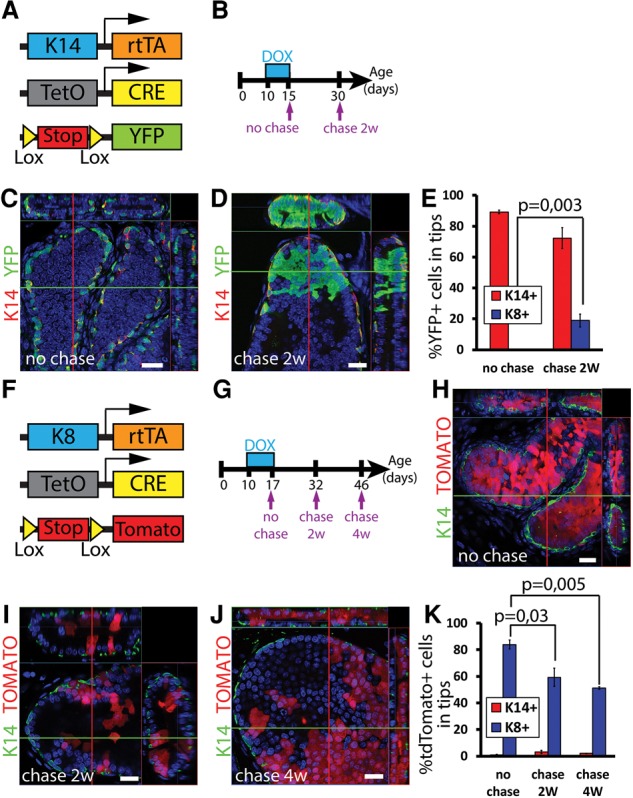
BCs contain multipotent SCs contributing to luminal expansion in the prostate. (*A*) Scheme summarizing the genetic strategy used to target YFP expression in K14rtTA/TetO-Cre/Rosa-YFP mice. (*B*) Scheme summarizing the protocol used to study the fate of cells targeted during prostate postnatal development using K14rtTA/TetO-Cre/Rosa-YFP mice. (*C*,*D*) Confocal imaging of immunostaining of K14 and YFP in the tip region at the end of DOX treatment (*C*) and 2 wk after induction (*D*) in 10-d-old K14rtTA/TetO-Cre/Rosa-YFP mice pulsed for 5 d with DOX. (*E*) Percentage of YFP^+^ cells in basal K14^+^ and luminal K8^+^ cells at the end of treatment and 2 wk after induction in the tip region in 10-d-old K14rtTA/TetO-Cre/Rosa-YFP mice pulsed for 5 d with DOX (40,925 and 43,395 cells out of four and three mice, respectively). (*F*) Scheme summarizing the genetic strategy used to target Tomato expression in K8rtTA/TetO-Cre/Rosa-tdTomato mice. (*G*) Scheme summarizing the protocol used to study the fate of cells targeted during prostate postnatal development using K8rtTA/TetO-Cre/Rosa-tdTomato mice. (*H*–*J*) Confocal imaging of immunostaining of K14 and fluorescent Tomato cells at the end of treatment (*H*), 2 wk after induction (*I*), and 4 wk after induction (*J*) in 10-d-old K8rtTA/TetO-Cre/Rosa-tdTomato mice pulsed for 7 d with DOX. (*K*) Percentage of Tomato^+^ cells in basal K14^+^ and luminal K8^+^ cells at the end of treatment and 2 wk after induction in the tip region in 10-d-old K8rtTA/TetO-Cre/Rosa-tdTomato mice pulsed for 7 d with DOX (26,687, 46,015, and 24,949 cells out of three, three, and two mice, respectively). (*C*,*D*,*H*,*I*,*J*) Orthogonal projections of 3D stacks. *P*-values in *E* and *K* represent a Student's *t*-test (paired, equal variances). Bars, 20 µm. Error bars indicate mean ± SEM. See Supplemental Table S6 for further information on statistics.

As we showed the existence of multipotent basal SCs in the prostate (Fig. 4), we performed lineage tracing at saturation experiments using the same experimental strategy as in the MG to assess the relative contribution of the flux of BCs to the luminal lineage during prostate postnatal development. Because most prostate postnatal branching takes place during the 2 wk after birth, DOX was administrated from P10 to K14rtTA/TetO-Cre/Rosa-YFP males for 5 d, and the fate of the labeled cells was assessed at the end of DOX treatment and 2 wk later (Fig. 7A,B). Confocal imaging of microdissected prostate WMs revealed that ∼90% of all K14-expressing cells expressed YFP after 5 d of DOX and that 0.3% of LCs were YFP^+^ (Fig. 7C,E; Supplemental Table S6, S10). Macroscopic analysis of WMs 2 wk after DOX treatment revealed that most of the changes in the labeled cells had taken place in the distal region of the ducts, called tips, rather than in the main ducts. For this reason, we quantified the number of fluorescent-positive cells in the tip region as well as in the whole prostate epithelium and the ducts (Fig. 7E; Supplemental Fig. S4C,D). After 2 wk of chase, ∼70% of BCs were still labeled. The bipotent basal SCs contributed 11% or 19% to the generation of LCs in the total epithelium and the tips, respectively, between P15 and P30 (*P*-value = 0.003) (Fig. 7E; Supplemental Tables S6, S10; Supplemental Fig. S4C), whereas, in the ductal part, this contribution only reached 1.7% (Supplemental Fig. S4A,B,D).

### Flux of cells from BCs that feeds prostate luminal lineage expansion

Using an independent approach, we next used the luminal lineage tracing at saturation in the prostate to estimate the flux of BCs to the luminal lineage, decreasing the proportion of LC-labeled cells during prostate development. DOX was administered to K8rtTA/TetO-Cre/Rosa-tdTomato P10 mice during a week (Fig. 7F,G). Confocal imaging of the microdissected prostate WM revealed that ∼85% of all K8-expressing cells expressed tdTomato after 7 d of DOX and that 1% of BCs were Tomato^+^ (Fig. 7J; Supplemental Tables S6, S10; Supplemental Fig. S4H), corresponding to BCs coexpressing K14 and K8, as previously described (Ousset et al. 2012). Similar to K14 experiments, macroscopic analysis of WMs after 2 and 4 wk of chase revealed that most of the changes in the labeled cells had taken place in the tip region rather than in the main ducts (Fig. 7F–J; Supplemental Tables S6, S10; Supplemental Fig. S4E–I). For this reason, we quantified the number of fluorescent-positive cells in all prostate epithelium as well as in the ducts and tip region separately at the different time points. The percentage of Tomato^+^ LCs dropped significantly from 85% to 66% and 65% at 2 and 4 wk, respectively, in the whole prostate (Supplemental Table S10; Supplemental Fig. S4H) and from 83% ± 3.4% to 59% ± 6.9% at 2 wk and 51% ± 1.1% at 4 wk in the tip region (*P*-value = 0.03 and 0.005) (Fig. 7J; Supplemental Table S6), showing that, from P17 to P32, 25% of the pool of LCs are generated by the differentiation of multipotent BCs. Interestingly, the percentage of Tomato^+^ LCs at 2 and 4 wk in the tip region is not significantly different, demonstrating that the switch from multipotency to unipotency of BCs occurs around P30. In contrast, the percentage of labeled LCs remained constant in the ductal part away from the tip region (Supplemental Table S10; Supplemental Fig. S4I).

## Discussion

Here, we showed that many CreER commonly used to target epithelial SCs are not entirely specific for a given lineage during development and homeostasis, complicating the interpretation of lineage tracing experiments to assess the multipotent fate of tissue-specific SCs. To assess cell fate in these tissues, we developed a novel statistical framework to assess with high precision and confidence the lineage relationship between different cell lineages, allowing the definition of the relative contributions of multipotent and unipotent tissue-specific stem and progenitor cells in the MG and prostate, and monitor in a quantitative manner the flux of cells transiting from one cell lineage to another. These two tissues present interesting similarities. They both originate from multipotent embryonic progenitors, which are replaced in adult tissue by unipotent basal and luminal SCs (Van Keymeulen et al. 2011; Choi et al. 2012; Ousset et al. 2012; Wang et al. 2013; Prater et al. 2014; Rodilla et al. 2015). The main difference between the prostate and the mammary BCs is the timing of the switch from multipotency to unipotency. Whereas, in the MG, this switch occurs rapidly before or after birth before pubertal expansion (Van Keymeulen et al. 2011), in the prostate, multipotent SC can be found until the late stage of prostate pubertal development (Ousset et al. 2012). Interestingly, prostate BCs expressing K5/K14 and K8 are found from the first postnatal days until the end of puberty (Ousset et al. 2012) and are enriched in multipotent progenitors. In this study, we initiated the tracing in the prostate at a different time point than in the MG to assess the fate of BCs during prostate postnatal development (which has been previously described) (Ousset et al. 2012; Pignon et al. 2013; Wang et al. 2014). If we had performed the tracing in the prostate at the same time point as in the MG, when the postnatal development of the prostate is largely completed, we would have assessed the fate of the unipotent SCs that sustain prostate adult homeostasis (Wang et al. 2009, 2013, 2014; Liu et al. 2011; Choi et al. 2012; Ousset et al. 2012; Lu et al. 2013; Pignon et al. 2013). Similarly, the dose of TAM in each system had been calibrated to induce clonal or chimeric targeting that differs between the MG and the prostate.

### Inferring multipotency from multicolor lineage tracing experiments

Conventional chimeric lineage tracing experiments using a monocolor reporter transgene (Rosa-YFP or Rosa-tdTomato) in the MG showed that many knock-in and transgenic CreER commonly used to mark epithelial SCs in different tissues, including Lgr5, Lgr6, K19, and Sox9, lead to the initial labeling of both BCs and LCs, although their relative proportion varies depending on the CreER. Lgr5 and Lgr6 preferentially marked BCs, while K19 and Sox9 preferentially marked LCs. However, a small proportion of LCs (for Lgr5 and Lgr6) or BCs (for K19 and Sox9) were initially labeled after a week, leading to the appearance of neighboring BCs and LCs labeled with the same fluorescent protein, giving the impression of multipotent SCs. Two parameters in these experiments (the initial labeling efficiency of individual isolated LCs far away from any labeled BC and the proportionate increase in the frequency of doublets at higher chimerism following a higher dose of TAM or DOX) should already suggest that colabeling of BCs and LCs may not represent the labeling of multipotent SCs but rather the initial labeling of neighboring unipotent cells.

To clarify these qualitatively ambiguous results, we developed a novel biostatistical framework to infer with high confidence the existence of multipotent and unipotent SCs in different epithelial tissues. By using WM confocal microscopy of multicolor clonal and chimeric lineage tracings and quantifying the relative frequency of recombination of each Confetti color, the overall induction frequency, the architecture of the tissue (how many LCs are in contact with one BC), and the specificity of the Cre, we could infer with high probability, irrespective of the cell proliferation rate, whether observed UPs arise from the labeling of a bipotent SC or occur by chance through recombination of two adjacent cells of different lineages. Using this method, we showed that the Cre used to support multipotency during MG development and homeostasis, although preferentially labeling BCs, also initially and independently labeled some LCs, as evidenced by the presence of isolated marked LCs just after Cre-mediated recombination and the fact that the frequency of these unicolor doublets was not statistically enriched over bicolor doublets. The frequency of these events corresponds to what would be observed by chance if the Cre were labeling two neighboring cells, further suggesting that MG development and adult remodeling are not mediated by multipotent SCs but rather by two independent lineages (Van Keymeulen et al. 2011; Lafkas et al. 2013; Prater et al. 2014; Tao et al. 2014; Rodilla et al. 2015). Different transgenic mice using a similar human K14 promoter fragment lead to very different targeting efficiency and specificity of labeling in the MG. The K14CreER mice generated by Vasioukhin et al. (1999) did not target the MG at all, whereas the K14rtTA transgene generated using the same promoter (Nguyen et al. 2006) targets the BC lineage of the MG very specifically and efficiently. The K14CreER transgenic mouse used by Rios et al. (2014), generated using a similar human K14 promoter fragment (Li et al. 2000), is less specific for the BC lineage and targets both BCs and LCs. These data demonstrate that the difference in the specificity and efficiency of cell targeting in the MG by different K14-driven inducible CREs reflects the transgenic nature of these mice, which is well known to lead to variation in transgene expression depending on the integration site in the mouse genome and is not related to intrinsic differences due to the promoter used.

In contrast to the situation found in MG development, during prostate development, our biostatistical analysis using a knock-in K5CreER mouse shows that UPs are overrepresented compared with BPs, clearly showing the existence of multipotent basal SCs during prostate development and demonstrating that the biostatistical framework developed here allowed us to discriminate between truly multipotent SCs and initial colabeling of two independent cell lineages. Further studies will need to be performed to assess whether the relative contribution of multipotent prostate SCs varies with the stage of development and the prostate lobes.

These results also demonstrate that one should be cautious in the interpretation of contiguous clusters of cells from different lineages labeled with the same color and that a correct assignation of clonal integrity requires rigorous, although straightforward, statistical analysis to infer multipotency versus unipotency with relatively few parameters. It is highly desirable that such variables are recorded in all future lineage tracing studies and that this approach becomes the standard for inferring multipotency in all tissues and organs in which new SCs and progenitors are identified.

### Lineage tracing at saturation defines the fate of all SCs and the flux of one cell lineage to another

Clonal and chimeric lineage tracing experiments can only draw firm and definitive conclusions about the cells labeled in each experiment and can potentially overlook the behavior of the cells that are not labeled by the CreER and would be maintained independently of these lineages. Only lineage tracing at saturation can resolve whether a small population of multipotent cells that would not be labeled by the chimeric lineage tracing really exists.

To be able to draw definitive conclusions about the cell fate of all cells within a given lineage, one needs to label all cells of this particular lineage. Transgenic and knock-in approaches using CreER can rarely achieve 100% recombination due to the relative toxicity and lethality of long-term and high-dose TAM administration. To label all BCs or all LCs from glandular epithelia, we used DOX-inducible systems, allowing us to mark with a very high efficiency almost all BCs or LCs in the prostate and the MG during development and adult homeostasis without apparent toxicity, and analyzed the fate of all BCs and LCs in these two tissues and the flux of cells that transit from one cell lineage to the other. Our data showed that, despite the complete labeling of all BCs of the MG during puberty, no LCs were labeled in adult virgin mice, demonstrating that all BCs are unipotent during this stage of development, as previously suggested by chimeric lineage tracing with K14-CreER, K5-CreER, Lgr5-CreER, Acta2-CreER, or Axin2-CreER (Van Keymeulen et al. 2011; de Visser et al. 2012; van Amerongen et al. 2012; Prater et al. 2014). The complete labeling of all BCs in adult virgin mice allowed the assessment of the contribution of BCs during adult remodeling occurring during pregnancy and lactation. Regardless of the technique used to assess the differentiation potential of BCs (FACS or 3D confocal analysis of WM MGs), we found that all BCs remained unipotent during the remodeling that accompanied pregnancy and lactation, showing the utility of lineage tracing at saturation and allowing the interrogation of the fate of each and every cell of a given tissue. The luminal lineage tracing at saturation during MG pubertal development clearly showed that LCs consist of self-sustained long-term self-renewing cells that are not replaced over time by multipotent BCs expressing K5 or any other cell type during adult remodeling but rather are replenished by their own pool of unipotent luminal SCs. Recent lineage tracings using Notch receptors have uncovered the existence of distinct SCs within the luminal lineage, sustaining ER/PR-positive and ER/PR-negative lineages independently (Rodilla et al. 2015). Further studies would be required to elucidate the cellular heterogeneity of the different unipotent luminal SCs within the MG.

Lineage tracing at saturation clearly identified the flux of cells from one compartment to another in a tissue in which multipotent SCs contribute to tissue development. In the prostate, labeling the BC lineage at saturation leads to the labeling of 90% of BCs and 19% of LCs in the tips 2 wk after the end of DOX administration. In contrast, when all LCs were labeled and then chased for 2 wk, the number of LCs labeled decreased by 25%, demonstrating the progressive dilution of labeled LCs by a flux of unlabeled cells coming from the basal multipotent SCs and showing the respective contribution of committed luminal progenitors and multipotent basal SCs to the overall development of the prostate.

### Conclusions

The techniques developed here can be used to decipher the lineage relationship in many different organs or tissues such as the stomach, the airway tracts, the MG, the prostate, and hematopoietic SCs, for which conflicting results have been described concerning the multipotency versus unipotency of their resident SCs (Bjerknes and Cheng 2002; Kim et al. 2005; Rawlins et al. 2009; Wang et al. 2009, 2013; Liu et al. 2011; Van Keymeulen et al. 2011; Choi et al. 2012; Lu et al. 2012, 2013; Sun et al. 2014). These techniques will also be useful in defining the cellular origin of cancers and the change in cell fate that accompanies cancer development, controlling tumor heterogeneity of the earliest step of tumor initiation (Choi et al. 2012; Blanpain 2013; Wang et al. 2013; Koren et al. 2015; Van Keymeulen et al. 2015).

## Materials and methods

### Mice

Lgr5-EGFP-IRES-CreER^T2^ (Barker et al. 2007), Lgr6-EGFP-IRES-CreER^T2^ (Snippert et al. 2010a), Rosa-YFP (Srinivas et al. 2001), and Rosa-tdTomato (Madisen et al. 2010) mice were obtained from the Jackson Laboratory. K14CreER^T2^ (Li et al. 2000) mice were provided by P. Chambon; Rosa-Confetti (Snippert et al. 2010b) mice were provided by H. Clevers, K14rtTA transgenic mice (Nguyen et al. 2006) were provided by Elaine Fuchs, TetO-Cre mice (Perl et al. 2002) were provided by Andreas Nagy, and Sox9CreER^T2^ mice (Kopp et al. 2011) were provided by Frédéric Lemaigre. The generation of K5CreER^T2^ (Van Keymeulen et al. 2011), K19CreER^T^ (Means et al. 2008), and K8rtTA (Watson et al. 2015) mice was described previously. All experimental mice used for experiments on breasts were female, and all experimental mice used for prostate experiments were male. All animals were mixed strains. No statistical methods were used to predetermine sample size. The experiments were not randomized. The investigators were not blinded to allocation during experiments and outcome assessment. Mice colonies were maintained in a certified animal facility in accordance with European guidelines. The experiments were approved by the local ethical committee (Comission d'Ethique et du Bien-Etre Animal [CEBEA]).

### Targeting Confetti, YFP, or tdTomato expression in the MG

For mosaic lineage tracing, Lgr5CreER^T2^/Rosa-tdTomato, Lgr6CreER^T2^/Rosa-tdTomato, K19CreER^T^/Rosa-Confetti, and Sox9CreER^T2^/Rosa-Confetti mice were induced with 15 mg (for Lgr5, Lgr6, K19; three injections of 5 mg every other day), 1.5 mg (for Lgr5), or 5 mg (for Sox9; one single injection) of TAM (Sigma) diluted in sunflower seed oil (Sigma) by intraperitoneal injection during puberty (4 wk) or adult homeostasis (8 wk), and the animals were killed 48 h or 1 wk after the last injection.

For multicolor tracing, K14CreER^T2^/Rosa-Confetti pubertal (4-wk-old) or adult (8-wk-old) mice were induced with 1.5 mg (for mosaic tracing), 0.05 mg (for clonal tracing in puberty), or 0.25 mg (for clonal tracing in adulthood) of TAM by intraperitoneal injection and killed 3 d or 1 or 4 wk later.

For lineage tracing at saturation, K14rtTA/TetO-Cre/Rosa-YFP or K8rtTA/TetO-Cre/Rosa-tdTomato female mice were induced by oral administration of 1 g/kg DOX food (BIO-SERV) or through 1 mg/mL drinking water (Sigma) during the whole course of pubertal development (3–8 wk ). K14rtTA/TetO-Cre/Rosa-YFP mice were analyzed after 3 d of DOX, at the adult stage (9- to 11-wk-old mice), during pregnancy, and during lactation. K8rtTA/TetO-Cre/Rosa-tdTomato mice were analyzed at the end of the DOX treatment (no chase), during pregnancy, and 20 wk after the end of the DOX treatment (20W chase).

### Analysis of cell proliferation

Cell proliferation was analyzed by immunofluorescence after pulsing K14CreER^T2^/Rosa-Confetti pubertal (4-wk-old) females with 50 mg/kg BrdU twice daily for 3 d.

### MG WM processing and immunostaining

For WM immunostaining, inguinal glands were dissected and enzymatically digested in Hank's balanced salt solution (HBSS) (Gibco) plus 300 U/mL collagenase (Sigma) and 300 µg/mL hyaluronidase (Sigma) for 30 min at 37°C with shaking. Glands were fixed in 4% PFA for 2 h at room temperature. Confetti samples were washed in ammonium chloride (0.5 M NH_4_Cl in PBS) for 20 min followed by three 10-min washes in PBS. For BrdU stainings, samples were treated with 1 N HCl for 30 min at 37°C with shaking and then processed the same way. Samples were incubated in blocking buffer (1% bovine serum albumin [BSA], 5% horse serum [HS], 0.8% Triton X in PBS) for 3 h at room temperature. The different primary antibody combinations were incubated overnight at room temperature and washed for 1 h at room temperature with PBS and 0.2% Tween20 before incubation with secondary antibodies diluted in blocking buffer at 1:800 for 5 h at room temperature. The following primary antibodies were used: anti-GFP (chicken, 1:800; Abcam, ab13970), anti-K14 (rabbit, 1:2000; Covance, PRB-155P-0100), anti-K14 (chicken, 1:2000; Covance, SIG-3476–0100), anti-K5 (rabbit, 1:1000; Covance, PRB-160P-0100), anti-E-cadherin (rat, 1:800; eBioscience, 14-3249-82), and anti-BrdU (rat, 1:200; Abcam, clone BU1/75, ab6326). The following secondary antibodies were used: anti-rabbit, anti-rat, and anti-chicken conjugated to Alexa fluor 488 (Molecular Probes), Rhodamine Red-X, or Cy5 (Jackson ImmunoResearch). Nuclei were stained with a Hoechst solution (1:1000 in PBS, 0.2% Tween20) for 30 min and washed for another hour in PBS and 0.2% Tween20 before mounting on slides in DAKO mounting medium supplemented with 2.5% Dabco (Sigma).

### MG immunofluorescent staining on sections

For pregnancy and lactation experiments, dissected MGs containing YFP or tdTomato were prefixed in 4% PFA for 2 h at room temperature. Tissues were washed three times in PBS for 5 min and incubated overnight at 4°Cin 30% sucrose in PBS. Tissues were embedded in OCT and kept at −80°C. Sections of 20 µm were cut using a HM560 Microm cryostat (Mikron Instruments). For immunofluorescence, tissue sections were incubated in blocking buffer (1% BSA, 5% HS, 0.8% Triton X in PBS) for 3 h at room temperature. The different primary antibody combinations were incubated overnight at 4°C. Sections were then rinsed three times for 30 min in PBS and incubated with the corresponding secondary antibodies diluted at 1:400 in blocking buffer for 5 h at room temperature. The following primary antibodies were used: anti-GFP (chicken, 1:1000; Abcam, ab13970), anti-K8 (rat, 1:500; Developmental Studies Hybridoma Bank, University of Iowa, Troma-I), anti-K14 (rabbit, 1:2000; Covance, PRB-155P-0100), anti-K14 (chicken, 1:2000; Covance, SIG-3476-0100), anti-K5 (rabbit, 1:1000; Covance, PRB-160P-0100), and anti-E-cadherin (rat, 1:800; eBioscience, 14-3249-82). The following secondary antibodies were used: anti-rabbit, anti-rat, and anti-chicken conjugated to Alexa fluor 488 (Molecular Probes), Rhodamine Red-X, or Cy5 (Jackson ImmunoResearch). Nuclei were stained with Hoechst solution (1:1000), and slides were mounted in DAKO mounting medium supplemented with 2.5% Dabco (Sigma).

### Mammary cell preparation

MGs were dissected, and the lymph nodes were removed before processing. Tissues were washed in HBSS and cut in 1-mm^3^ pieces with scissors. Minced tissues were placed in HBSS plus 300 U/mL collagenase (Sigma) plus 300 mg/mL hyaluronidase (Sigma) and digested for 2 h at 37°C with shaking. EDTA at a final concentration of 5 mM was added for 3 min to the resultant organoid suspension, followed by 0.25% Trypsin-EDTA for 1 min before filtration through a 40-µm mesh, two successive washes in 2% FBS/PBS, and labeling.

### Cell labeling and flow cytometry

Two million to 5 million cells per condition were incubated in 250 µL of 2% FBS/PBS with fluorochrome-conjugated primary antibodies for 30 min with shaking every 10 min. Primary antibodies were washed with 2% FBS/PBS, and cells were resuspended in 2.5 mg/mL DAPI (Invitrogen) before analysis. The following primary antibodies were used for the analysis of K14rtTA/TetO-Cre/Rosa-YFP mice: PECy7-conjugated anti-CD24 (1:100; BD Biosciences, clone M1/69), APC-conjugated anti-CD29 (1:100; eBiosciences, clone eBioHMb1-1), PE-conjugated anti-CD45 (1:100; eBiosciences, clone 30-F11), PE-conjugated anti-CD31 (1:100; BD Biosciences, clone MEC 13.33), and PE-conjugated anti-CD140a (1:100; eBiosciences, clone APA5). Data analysis was performed on a BD LSR Fortessa using FACS DiVa software (BD Biosciences). Dead cells were excluded with DAPI; CD45^+^, CD31^+^, and CD140a^+^ cells were excluded (Lin^+^) before analysis of the YFP^+^ cells. Primary antibodies used for the analysis of K8rtTA/TetO-Cre/Rosa-tdTomato mice were PECy7-conjugated anti-CD24 (1:100; BD Biosciences, clone M1/69), FITC-conjugated anti-CD29 (1:100; BD Biosciences, clone Ha2/5), APC-conjugated anti-CD45 (1:100; eBiosciences, clone 30-F11), APC-conjugated anti-CD31 (1:100; eBiosciences, clone 390), and APC-conjugated anti-CD140a (1:100; eBiosciences, clone APA5). A minimum of five 10^5^ total events per mouse were analyzed.

### Targeting Confetti, YFP, and tdTomato expression in the prostate

For clonal lineage tracing induced at birth, K5CreER^T2^/Rosa-Confetti mice were induced with 0.1 mg of TAM by intraperitoneal injection and killed 10 d later. For lineage tracing at saturation, K14rtTA/TetO-Cre/Rosa-YFP or K8rtTA/TetO-Cre/Rosa-tdTomato mice were induced at P10 with 1 mg of DOX intraperitoneally (diluted in PBS) and maintained on DOX treatment for 5 or 7 d for K14rtTA/TetO-Cre/Rosa-YFP or K8rtTA/TetO-Cre/Rosa-tdTomato, respectively, through DOX provided to the mother in the drinking water (2 mg/mL) and food (10 g/kg) (Bio-Serv). The mice were then killed at the end of DOX treatment (no chase), 2 wk later (chase 2W), or 4 wk later (chase 4W).

### Prostate WM processing and immunostaining

Samples processed at P10 were obtained by dissection of the entire urogenital system followed by removal of the fat and bladder tissue. The final WM samples contained the whole prostate tissue as well as the urethra and seminal vesicles. For all samples processed at later time points, prostate tissue was initially dissected and then microdissected to isolate the different lobes (ventral, dorso–lateral, and anterior). The epithelial tissue was then digested in a solution of 1% collagenase (diluted in HBSS) (Sigma) for 3–8 min depending on the age. Tissues were then prefixed 2 h in 4% PFA at room temperature and washed twice with PBS for 10 min. WMs were incubated in blocking buffer (1% BSA, 5% HS, 0.8% Triton X in PBS) for 3 h at room temperature. The different primary antibody combinations were incubated overnight at room temperature in blocking buffer. WMs were then rinsed three times for 10 min in PBS and 0.2% Tween and incubated with proper secondary antibodies diluted at 1:400 in blocking buffer for 5 h at room temperature. The following primary antibodies were used: anti-GFP (chicken, 1:1000; Abcam, ab13970) and anti-K14 (rabbit, 1:2000; Covance, PRB-155P-0100). The following secondary antibodies were used: anti-rabbit and anti-chicken conjugated to Alexa fluor 488 (Molecular Probes), Rhodamine Red-X, or Cy5 (Jackson ImmunoResearch). Nuclei were stained in Hoechst solution (diluted in 0.2% PBS, 1:1000 Tween20 for YFP and tdTomato mice or 1:10,000 for Confetti mice) incubated for 30 min (YFP and tdTomato mice) or 2 h (Confetti mice). Tissues were then rinsed twice for 10 min in PBS and 0.2% Tween20 and mounted on slides in DAKO mounting medium supplemented with 2.5% Dabco (Sigma). For the analysis performed in the tip region, tips were defined as the most distal 250 µm of the ducts.

### Microscope image acquisition

Confocal images were acquired at room temperature using a Zeiss LSM780 confocal microscope fitted on an Axiovert M200 inverted microscope equipped with a C-Apochromat (40×, NA = 1.2) water immersion objective (Carl Zeiss, Inc.). Optical sections of 1024 × 1024 pixels were collected sequentially for each fluorochrome. The data sets generated were merged and displayed with ZEN software.

### Statistical analysis

To rigorously test whether the observed frequencies of UPs in a given tissue reflect the clonal expansion of induced bipotent BCs or can be explained by the chance independent labeling of BCs and LCs, we employed a simple biostatistical modeling scheme together with statistical hypothesis testing. Based on the idea that unipotency and bipotency should be distinguishable in the fraction of UPs among all observed pairs, in the following, we calculated the probability of finding their experimentally observed frequencies or any higher frequency (which together define the *P*-value). We found that the frequency of observed UPs is in agreement with by chance labeling in the MG (*P* = 0.65), while the experimental data for the prostate suggest bipotent cell divisions (*P* = 0.0064). Indeed, we show that a simple stochastic model comprising random induction can quantitatively predict the empirical fraction of UPs and the overall frequency of pairs in the MG, while these doublets are overrepresented in the prostate. This biostatistical model provides a simple framework to evaluate the evidence for multipotency in tracing data.

We defined a UP event as a BC touching an LC of the same color in a MG. In the developing prostate, despite a clonal induction frequency, we observed frequent unicolor groups of cells in the basal and luminal layer, indicating a significant amount of cell divisions between labeling and analysis. As these cohesive patches are clonal with high probability, we consider touching groups of (unicolor) cohesive patches in the basal and luminal layer as (unicolor) pairs. For simplicity, in the following, we make use of the terminology for single cells (as in the MG), but keep in mind that, in the prostate, we are potentially referring to groups of adjacent cells. In the first part of our analysis, we focused on the fraction of UPs among all observed pairs. Note that, since our analysis was conditioned on the total number of pairs, the fraction of UPs should not depend on the overall induction frequencies and should not be affected by spatial inhomogeneity or cell divisions within the basal layer. For now, we therefore pool-induced clones in all regions in the tissue. To analyze the confetti labeling data, let us begin by defining the probability that, following TAM administration, a BC or LC is induced. Of course, this probability may vary according to the specific color of the fluorescent reporter gene as well as mouse to mouse. We therefore consider induction separately for each mouse and each of the four Confetti colors.

Let us suppose that a given frame of tissue has a total of *B* BCs, of which *b*_*C*_ are fluorescently labeled in a given color, *C*, and *L* LCs, of which *l*_*C*_ are labeled in that color. We may then define β_*C*_ = *b*_*C*_/*B* as the probability that, following TAM administration, a BC is induced with color *C* (one of the four Confetti colors). The respective probabilities for LCs are defined by λ_*C*_ = *l*_*C*_/*L*. We now define the probability that the BC and LC in a given pair are labeled in the same color:

$$\nu =\underset{C}{\sum }{\bar{\beta }}_{C}{\bar{\lambda }}_{C},$$

where ${\bar{\beta }}_{C}=\;\beta_{C}/\sum_{C^{\prime }}\beta_{C^{\prime }}$ and ${\bar{\lambda }}_{C}=\lambda_{C}/\sum_{C^{\prime }}\lambda_{C^{\prime }}$ are the relative induction probabilities of the four Confetti colors (for experimental values, see Tables 1, 2). If the induction of LCs is statistically independent from the induction events of neighboring BCs, the total number of UPs, *u*, among all pairs, *s*, follows a binomial distribution:

$$P(u)=\frac{s!}{u!(s-u)!}\nu^{u}{\left(1-\nu \right)}^{s-u}.$$

With this result, we are already in a position to perform a statistical test on the hypothesis that the experimental data are the result of pure chance. The experimental data would not arise from chance if BCs were bipotent. Since bipotency would lead to an increase in the number of UPs, we consider positive deviations as potentially significant (one-tailed test). If u* is the experimentally observed number of UPs, then the probability of finding u* or more UPs is called the *P*-value, and it is in our case given by

$$p=\sum_{u=u*}^{\infty }P(u).$$

It is common scientific practice that results corresponding to a *P*-value of <0.05 are considered statistically significant. In contrast, experimental data corresponding to a *P*-value >0.05 are commonly considered consistent with the hypothesis that the experimental outcome is a result of chance. We calculated these *P*-values separately for each mouse and combined these probabilities to a single *P*-value, making use of Fisher's method. The resulting *P*-value is 0.65 for the MG and 0.0064 for the prostate. This already clearly indicates that there is no reason to assume bipotency based on the observed frequencies of UPs in the MG. In contrast, the frequency of UPs in the prostate cannot be explained by random labeling alone, strongly suggesting a bipotent basal population.

**Table 1.**
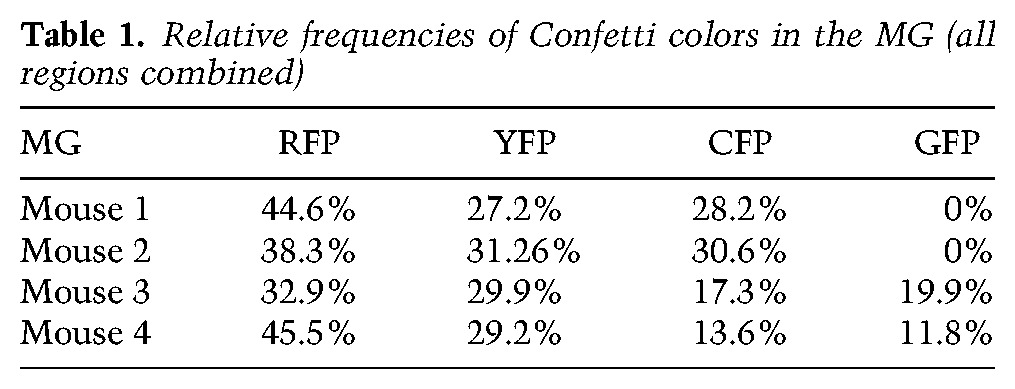
Relative frequencies of Confetti colors in the MG (all regions combined)

**Table 2.**
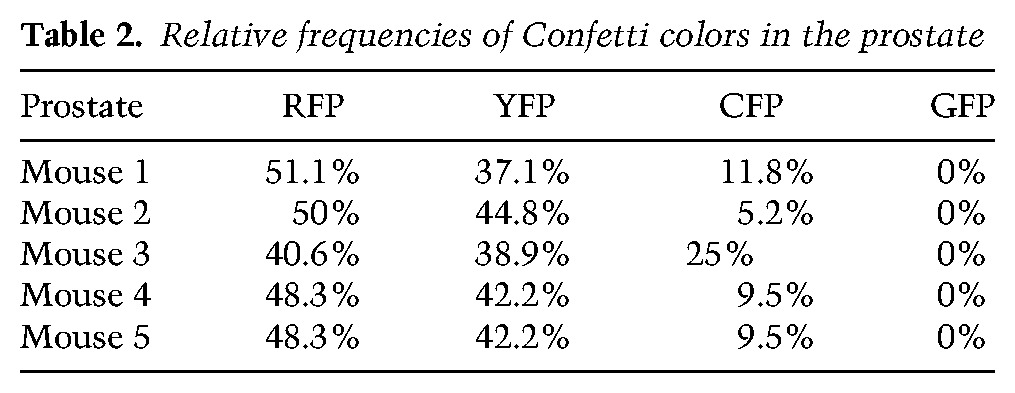
Relative frequencies of Confetti colors in the prostate

To further test whether the results are in agreement with stochastic labeling, we went one step further and asked whether our simple stochastic model is able to quantitatively predict the experimental data in both tissues. If this were the case, the probability of finding a UP (*v*) should be equal to the observed fraction of UPs. Indeed, the theoretical prediction is in excellent agreement with the experimental value for all mice in the MG (Fig. 3P), further supporting the hypothesis that BCs in this tissue are unipotent. For the prostate, we found consistently more UPs than predicted by random labeling, suggesting that this excess stems from BC divisions giving rise to a luminal daughter cell. Note that the above calculations depend only on the relative probabilities of the four Confetti colors. Furthermore, by considering the conditional probability for a given total number of pairs composed of one BC and one LC (or one basal clone and one luminal clone), we could circumvent potentially ambiguous factors such as the overall induction frequency, which is spatially inhomogeneous in the MG or the tissue architecture (see below). Calculating the *P*-value based on the nonconditional number of UPs (i.e., including BPs and nonpaired cells), we would have to take these factors into account, potentially leading to artificially low *P*-values.

Having calculated the number of UPs for a given number of pairs, we sought to describe the overall number of pairs. To this end, we had to take into account the induction probabilities and the geometric arrangement of BCs and LCs. In the MG, the induction is inhomogeneous, potentially due to differential blood supplies, depending on the region. In our statistical analysis, we took these differences explicitly into account. For the MG, we therefore sorted the image frames into regions of high induction and regions of low induction and considered induction within each image as homogeneous and random. Following induction in the MG, we assumed that only a small portion of marked cells had time to undergo even one round of division prior to fixation of tissue and analysis; the probability that a marked BC of color *C* is induced in proximity to unmarked neighbors in the luminal layer can be approximated by

$$\sigma_{C|z}={\left(1-\underset{C^{\prime }}{\sum }\lambda_{C^{\prime }}\right)}^{z}\beta_{C},$$

where *z* is the effective coordination, defined as the number of LCs that are proximate to any given BC.

In the prostate, we frequently observed BCs touching more than one LC of the same color. As in the MG, pairs of multiple LCs are much more likely to arise by proliferation than by chance labelling, we scored all of these events as “UPs.” We therefore obtained the same formula for σ_*C/z*_ in the prostate as in the MG.

Similarly, the probability that a marked BC of color C1 is induced in proximity to a single LC of color C2 is given by

$$\sigma_{C1,C2|z}=z{\left(1-\underset{C2^{\prime }}{\sum }\lambda_{C2^{\prime }}\right)}^{z-1}\lambda_{C2}\beta_{C1}.$$

Here, having in mind a relatively low frequency of BC induction, we neglected the chance of merger of two neighboring events; i.e., where two neighboring marked BCs shared a common luminal neighbor. The number of neighbors, *z*, is not entirely homogeneous, as we found BCs neighboring between three and seven LCs in the MG and between one and three cells in the prostate. To correctly take the variability in coordination into account, we counted the numbers of neighbors for 40 cells (the MG) or 15 cells (the prostate) (Table 3) and, from these data, calculated the empirical distribution of *z*, *F*(*z*). The overall probability that a BC labeled in color C1 is paired to a LC labeled in color C2 is given by

$$\sigma_{C1,C2}=\lambda_{C2}\beta_{C1}\underset{z}{\sum }z{\left(1-\underset{C2^{\prime }}{\sum }\lambda_{C2^{\prime }}\right)}^{z-1}F(z).$$

Therefore, the conditional probability that a given labeled BC neighbors a LC of color *C* is given by σ_*C*_ = σ_*C1,C*_/β_*C1*_. Finally, summation over all colors gives the probability that a given BC is paired:

$$\sigma =\left(\underset{C}{\sum }\lambda_{C}\right)\underset{z}{\sum }z{\left(1-\underset{C^{\prime }}{\sum }\lambda_{C^{\prime }}\right)}^{z-1}F(z).$$

If the total luminal induction probability $\left(\underset{C}{\sum }\lambda_{C}\right)$ and variability in coordination are small, we can expand this expression to the first order to obtain an approximation for clonal tissues: $\sigma \approx \bar{z}\underset{C}{\sum }\lambda_{C}$. In this case, the fraction of paired BCs is given by the average coordination,$\;\bar{z}$, times the overall induction probability of LCs.

**Table 3.**
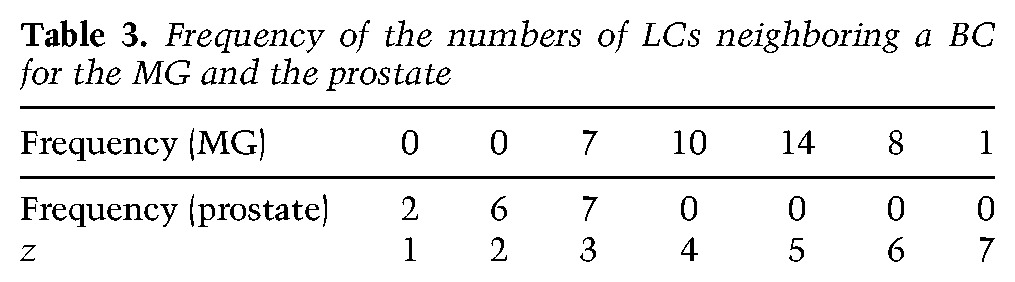
Frequency of the numbers of LCs neighboring a BC for the MG and the prostate

Therefore, since LC induction events are statistically independent, the probability of finding *s* pairs if *B* BCs were labeled in a given frame of tissue is again binomially distributed:

$$P(s)=\left(\begin{array}{c} B\\ s \end{array}\right)\sigma^{s}{\left(1-\sigma \right)}^{B-s}.$$

Note that σ is dependent on the variation in induction frequency between frames in the MG (taken as high and low) and among different mice. To combine the results from different areas of tissue in the MG, we note that the binomial distribution is well approximated by a normal distribution if the number of BCs, *B*, and the number of pairs, *s*, are sufficiently high. In this case, summing up the numbers of pairs in highly (*H*) and lowly (*L*) induced regions yields a normal distribution with mean σ_*H*_*B*_*H*_ + σ_*L*_*B*_*L*_, and, consequently, the fraction of paired BCs is given by

$$\bar{\sigma }=\frac{\sigma_{H}B_{H}+\sigma_{L}B_{L}}{B_{H}+B_{L}}.$$

With this definition, we found that the overall number of pairs again follows a binomial distribution:

$$P(\bar{s})=\left(\begin{array}{c} B_{H}+B_{L}\\ \bar{s} \end{array}\right){\bar{\sigma }}^{\bar{s}}{\left(1-\bar{\sigma }\right)}^{B_{H}+B_{L}-\bar{s}}.$$

Comparing the predicted frequencies of pairs in each mouse, $\bar{\sigma }$, with the measured frequencies, we again found an excellent agreement with our simple stochastic model for the MG (Fig. 3Q), while, in the prostate, we found consistently more pairs than expected by chance (Fig. 4I). Note that the color balance is variable between mice. However, this does not affect the validity of our results for the following reason: To calculate the theoretical prediction for the fraction of UPs and the fraction of paired BCs, we calculated the color balance and the induction frequency separately for each mouse. In the MG, where induction was spatially inhomogeneous, we additionally performed the calculations separately for high and low induction regions. The only parameter that was taken representatively for all mice for both the MG and prostate was the tissue architecture. To combine results for different regions and calculate the *P*-value, we made use of properties of the binomial distribution. At no point in the calculations did we average over different mice such that we took mouse-to-mouse heterogeneity fully and correctly into account.

In summary, we developed a simple statistical framework involving random labeling and statistical hypothesis testing to test whether the observation of UPs of BCs and LCs in a given tissue does imply bipotency. In fact, we found that our simple model can accurately predict both the frequencies of UPs and the total frequency of pairs in the MG, while both quantities are consistently overrepresented in the prostate. Taken together, our statistical analysis provides sound evidence that BCs in the MG are unipotent, while BCs in the prostate are bipotent. We emphasize that this simple framework can also be used to test for multipotency in parallel biological contexts.

## Supplementary Material

Supplemental Material
