# Supplementary material for: Quantitative lineage tracing strategies to resolve multipotency in tissue-specific stem cells
Source: Genes Dev. 2016 Jun 1;30(11):1261–77. doi: 10.1101/gad.280057.116 (PMC4911926; doi:10.1101/gad.280057.116)
Supplement: Supplemental Material [file supp_30_11_1261__index.html]

Quantitative lineage tracing strategies to resolve multipotency in tissue-specific stem cells — Quantitative lineage tracing strategies to resolve multipotency in tissue-specific stem cells — Supplemental Material 

# Quantitative lineage tracing strategies to resolve multipotency in tissue-specific stem cells

## Supplemental Material

- Supplemental\_Figures\_Tables.pdf
