# Supplementary material for: Quantitative lineage tracing strategies to resolve multipotency in tissue-specific stem cells
Source: Genes Dev. 2016 Jun 1;30(11):1261–77. doi: 10.1101/gad.280057.116 (PMC4911926; doi:10.1101/gad.280057.116)
Supplement: Supplemental Material [file supp_gad.280057.116_Supplemental_Figures_Tables.pdf]

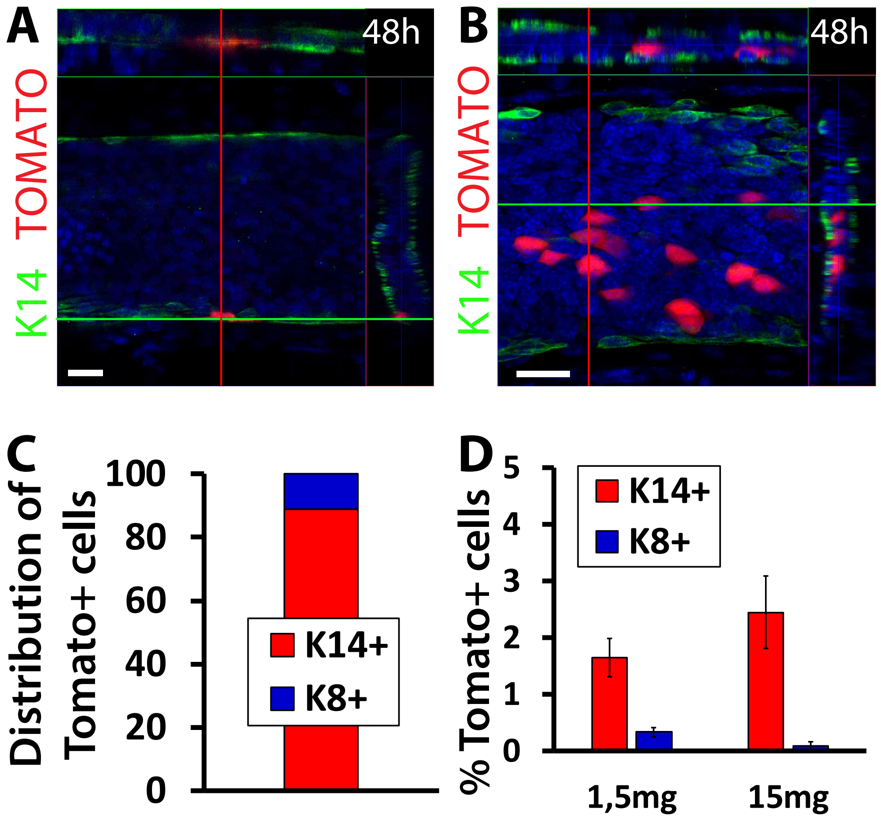

Supplemental Figure S1. Lgr5CreER^{T2} targets initially and independently unipotent BCs and LCs in the MG

(A, B) Confocal imaging of immunostaining of K14 and Tomato 48 hours after TAM administration (1,5mg) in adulthood to Lgr5CreER^{T2}/Rosa-Tomato mice. (A) shows isolated BCs, (B) shows isolated LCs. (C) Graph representing the distribution of basal K14+ and luminal K8+ cells among Tomato+ cells 48 hours after induction in adult mice. (D) Graph representing the chimerism (percentage of labeled BCs and LCs) 1 week after induction with 1,5mg or 15mg TAM in adult mice. (A, B) represent orthogonal projections of 3D stacks. Scale bars, 20 μm; error bars indicate mean± s.e.m. See Supplemental Table 7 for further information on statistics.

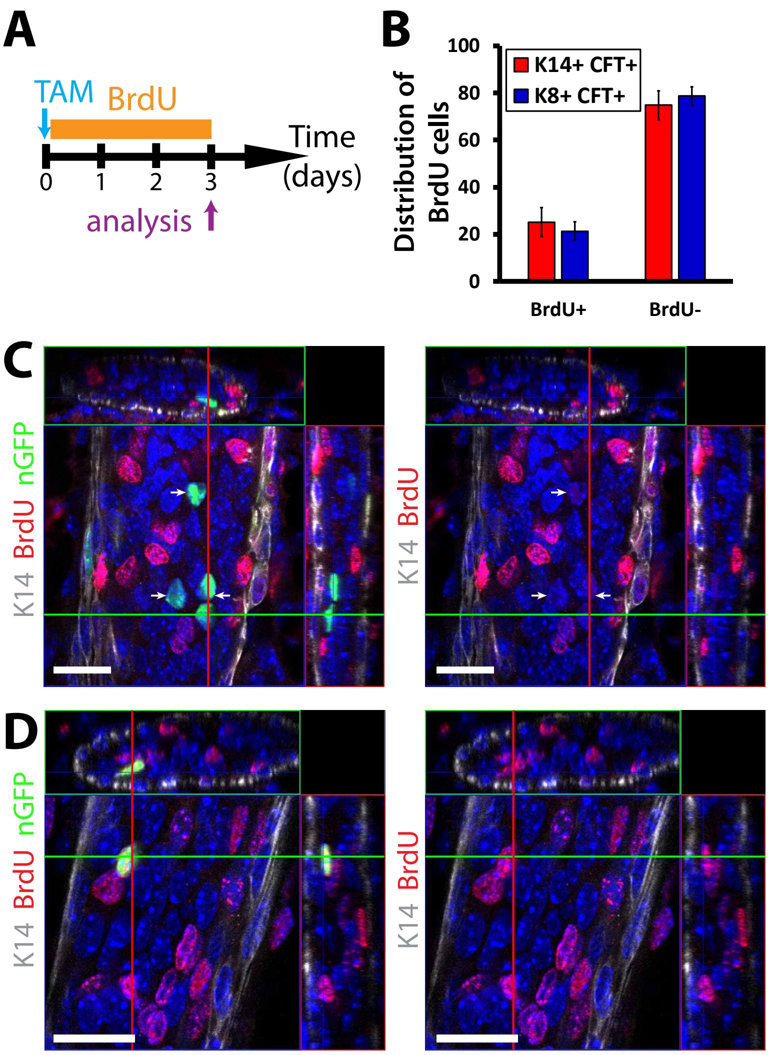

Supplemental Figure S2. K14CreER^{T2} targets initially and independently unipotent BCs and LCs in the MG

(A) Scheme summarizing the protocol used to study the fate of cells targeted at puberty using K14CreER^{T2}/Rosa-Confetti mice. (B) Graph representing the distribution of BrdU cells among K14+CFT+ and K8+CFT+ cells 3 days after induction in puberty (426 cells out of 3 mice). (C-D) Confocal imaging of immunostaining of K14, BrdU and fluorescent Confetti cells 3 days after TAM administration (1,5mg) at puberty in K14CreER^{T2}/Rosa-Confetti mice. (C) shows isolated LCs negative for BrdU (arrows), (D) shows isolated LCs positive for BrdU. (C, D) represent orthogonal projections of 3D stacks. Scale bars, 20 µm; error bars indicate mean± s.e.m. See Supplemental Table 8 for further information on statistics.

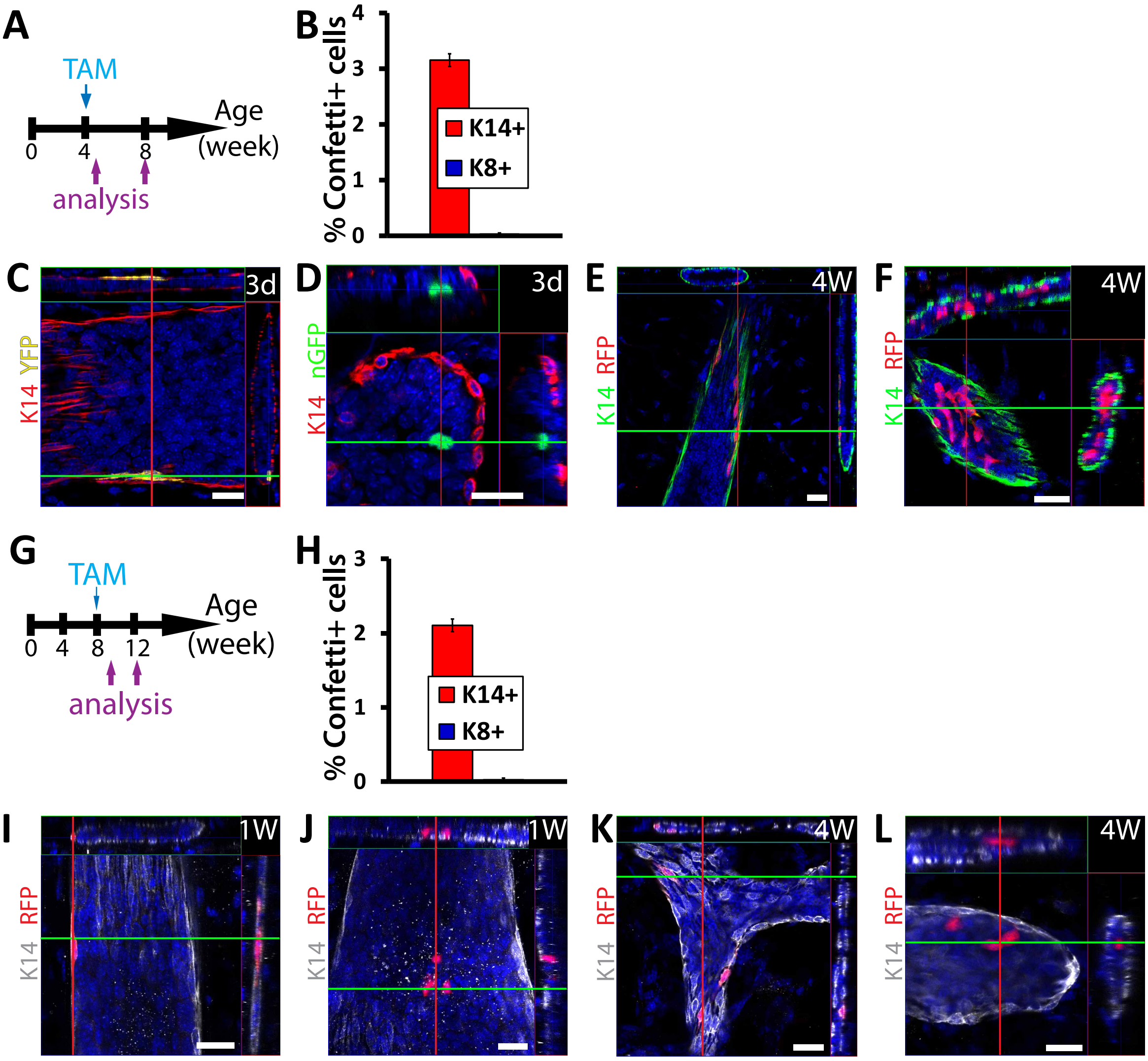

Supplemental Figure S3. K14CreER^{T2} targets initially and independently unipotent BCs and LCs in the MG

(A) Scheme summarizing the protocol used to study the fate of cells targeted at puberty using K14CreER^{T2}/Rosa-Confetti mice. (B) Graph representing the chimerism (percentage of labeled BCs and LCs) 3 days after induction in puberty (55887 cells out of 3 mice). (C-F) Confocal imaging of immunostaining of K14 and fluorescent Confetti cells 3 days (C, D) and 4 weeks (E, F) after TAM administration (0,05mg) at puberty in K14CreER^{T2}/Rosa-Confetti mice. (G) Scheme summarizing the protocol used to study the fate of cells targeted in adulthood using K14CreER^{T2}/Rosa-Confetti mice. (H) Graph representing the chimerism (percentage of labeled BCs and LCs) 1 week after induction in adult mice (45980 cells out of 2 mice). (I-L) Confocal imaging of immunostaining of K14 and fluorescent Confetti cells 1 week (I, J) and 4 weeks (K, L) after TAM administration (0,25mg) in adulthood in K14CreER^{T2}/Rosa-Confetti mice. (C-F, I-L) represent orthogonal projections of 3D stacks. Scale bars, 20 µm; error bars indicate mean± s.e.m. See Supplemental Table 9 for further information on statistics.

Wuidart_FigS4

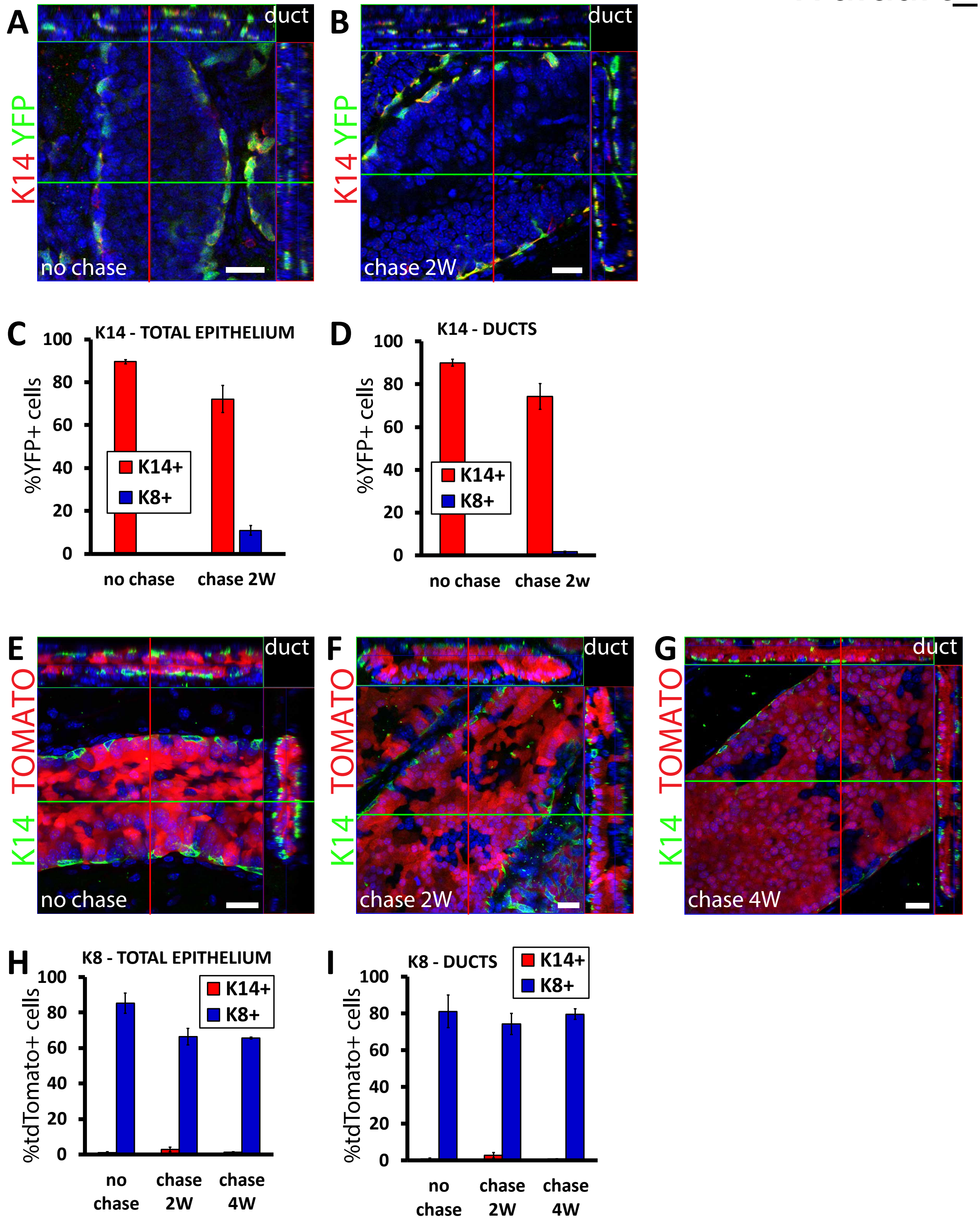

Supplemental Figure S4. Basal cells contain multipotent SCs contributing to luminal expansion in the prostate

(A, B) Confocal imaging of immunostaining of K14 and YFP in prostate ducts at the end of DOX treatment (A) and 2 weeks after induction (B) in 10 days old K14rtTA/TetO-Cre/Rosa-YFP mice pulsed for 5 days with DOX. (C, D) Percentage of YFP+ cells in basal K14+ and luminal K8+ cells at the end of DOX treatment (no chase) and 2 weeks after induction in the whole prostate epithelium (C) and in the prostate main ducts (D) in 10 days old K14rtTA/TetO-Cre/Rosa-YFP mice pulsed for 5 days with DOX. (E-G) Confocal imaging of immunostaining of K14 and fluorescent Tomato cells at the end of treatment (E), 2 weeks after induction (F) and 4 weeks after induction (G) in 10 days old K8rtTA/TetO-Cre/Rosa-tdTomato mice pulsed for 7 days with DOX. (H, I) Percentage of Tomato+ cells in basal K14+ and luminal K8+ cells at the end of treatment, 2 weeks after induction and 4 weeks after induction in the whole prostate epithelium (H) and in the prostate main ducts (I) in 10 days old K8rtTA/TetO-Cre/Rosa-tdTomato mice pulsed for 7 days with DOX . (A, B, E-G) represent orthogonal projections of 3D stacks. Scale bars, 20 µm; error bars indicate mean± s.e.m. See Supplemental Table 10 for further information on statistics.

Supporting data for Figure 1G – Lgr5-EGFP-IRES-CreER^{T2}/Rosa-tdTomato – 1,5mg TAM - Adulthood					
**1w Mouse**	**K14+ TOM+**	**% K14+ in TOM+**	**K8+ TOM+**	**% K8+ in TOM+**	**Total TOM+ cells**
**#1**	703	77,3	206	22,7	909
**#2**	265	42,7	356	57,3	621
**#3**	852	63,8	483	36,2	1335
	**Mean**	**61,3**	**Mean**	**38,7**	

Supporting data for Figure 1N– Lgr6-EGFP-IRES-CreER^{T2}/Rosa-tdTomato - 15mg TAM - Adulthood					
**1w Mouse**	**K14+ TOM+**	**% K14+ in TOM+**	**K8+ TOM+**	**% K8+ in TOM+**	**Total TOM+ cells**
**#1**	965	58,9	673	41,1	1683
**#2**	908	66,7	453	33,3	1361
**#3**	967	57,4	717	42,6	1684
	**Mean**	**61**	**Mean**	**39**	

Supporting data for Figure 2G – K19CreER^{T}/Rosa-Confetti -15mg TAM – Adulthood					
**1w Mouse**	**K8+ CFT+**	**% K8+ in CFT+**	**K14+ CFT+**	**% K14+ in CFT+**	**Total CFT+ cells**
**#1**	457	97,4	12	2,6	469
**#2**	544	97,8	12	2,2	556
**#3**	244	96,9	8	3,2	252
	**Mean**	**97,4**	**Mean**	**2,6**	

Supporting data for Figure 2N – Sox9CreER^{T2}/Rosa-Confetti - 5mg TAM - Adulthood					
**1w Mouse**	**K8+ CFT+**	**% K8+ in CFT+**	**K14+ CFT+**	**% K14+ in CFT+**	**Total CFT+ cells**
**#1**	1822	99,9	1	0,1	1823
**#2**	1390	92,9	106	7,1	1496
**#3**	881	93,8	58	6,2	939
	**Mean**	**95,6**	**Mean**	**4,4**	

Supporting data for Figure 3C-3D – K14CreER^{T2}/Rosa-Confetti – 1,5mg TAM - Puberty								
**3d Mouse**	**K14+ CFT+**	**% K14+ in CFT+**	**K8+ CFT+**	**% K8+ in CFT+**	**K14+**	**% CFT+ in K14+**	**K8+**	**% CFT+ in K8+**
**#1**	214	78,4	59	21,6	621	34,5	1863	3,2
**#2**	304	84,7	55	15,3	1188	25,6	3563	1,5
**#3**	158	55,8	125	44,2	2072	7,9	6217	2,1
	**Mean**	**73**	**Mean**	**27**	**Mean**	**22,7**	**Mean**	**2,3**
					**SEM**	**7,8**	**SEM**	**0,5**

Supporting data for Figure 3M-3N – K14CreER^{T2}/Rosa-Confetti -1, 5mg TAM - Adulthood								
**1w Mouse**	**K14+ CFT+**	**% K14+ in CFT+**	**K8+ CFT+**	**% K8+ in CFT+**	**K14+**	**% CFT+ in K14+**	**K8+**	**% CFT+ in K8+**
**#1**	105	49,3	108	50,7	2102	5	6306	1,7
**#2**	1099	76,3	341	23,7	2601	42,3	7803	4,4
**#3**	534	85,7	89	14,3	3695	14,5	11085	0,8
**#4**	745	91,3	71	8,7	3176	23,5	9528	0,7
	**Mean**	**75,7**	**Mean**	**24,3**	**Mean**	**21,3**	**Mean**	**1,9**
	**SEM**	**7,9**	**SEM**	**0,9**				

Supporting data for Figure 3O – K14CreER^{T2}/Rosa-Confetti – 1,5mg TAM - Adulthood									
**1w Mouse**	**Basal patches**	**%**	**Luminal patches**	**%**	**UPs**	**%**	**BPs**	**%**	**Total**
**#1**	93	47	93	47	4	2	8	4	198
**#2**	640	67,4	71	7,5	76	8	162	17,1	949
**#3**	473	85,4	57	10,3	5	0,9	19	3,4	554
**#4**	580	89,9	36	5,6	14	2,2	15	2,3	645
	**Mean**	**76,6**	**Mean**	**11**	**Mean**	**4,1**	**Mean**	**8,3**	**100**

Supporting data for Figure 4C – K5CreER^{T2}/Rosa-Confetti – 0,1mg TAM – Induction P10				
**10d Mouse**	**K14+ CFT+**	**% K14+ in CFT+**	**K8+ CFT+**	**% K8+ in CFT+**
**#1**	173	64,8	94	35,2
**#2**	145	53,3	127	46,7
**#3**	105	80,8	25	19,2
**#4**	201	72,8	75	27,2
**#5**	263	81,7	59	18,3
	**Mean**	**70,7**	**Mean**	**29,3**

Supporting data for Figure 4G – K5CreER^{T2}/Rosa-Confetti – 0,1mg TAM – Induction P10									
**10d Mouse**	**Basal patches**	**%**	**Luminal patches**	**%**	**UPs**	**%**	**BPs**	**%**	**Total**
**#1**	104	71,7	39	26,9	2	1,4	0	0	145
**#2**	83	60,6	49	35,8	5	3,6	1	0,7	137
**#3**	80	85,1	12	1,8	2	2,1	0	0	94
**#4**	129	74,6	42	24,3	2	1,2	0	0	173
**#5**	188	83,6	33	14,8	4	1,8	0	0	225
	**Mean**	**75,1**	**Mean**	**22 ,9**	**Mean**	**3,6**	**Mean**	**0,7**	**100**

Supporting data for Figure 5H-5I – K14rtTA/TetO-Cre/Rosa-YFP								
**3d Mouse**	**YFP+ K14+**	**% K14+ in YFP+**	**YFP+ K8+**	**% K8+ in YFP+**	**K14+**	**% YFP+ in K14+**	**K8+**	**% YFP+ in K8+**
**#1**	338	98,3	6	1,7	798	43,3	2393	0,2
**#2**	402	99,5	2	0,5	823	46,6	2468	0,1
**#3**	554	100	0	0	1616	33,5	4848	0
	**Mean**	**99,3**	**Mean**	**0,75**	**Mean**	**41,1**	**Mean**	**0,1**
	**SEM**	**0,5**	**SEM**	**0,5**	**SEM**	**3,9**	**SEM**	**0,07**
**6W Mouse**	**YFP+ K14+**	**% K14+ in YFP+**	**YFP+ K8+**	**% K8+ in YFP+**	**K14+**	**% YFP+ in K14+**	**K8+**	**% YFP+ in K8+**
**#1**	894	99,8	2	0,2	932	95,9	2796	0,1
**#2**	1029	99,7	3	0,3	1065	96,5	3194	0,08
**#3**	964	99,9	1	0,1	969	99,6	2390	0,05
	**Mean**	**99,8**	**Mean**	**0,2**	**Mean**	**97,3**	**Mean**	**0,08**
	**SEM**	**0,05**	**SEM**	**0,05**	**SEM**	**1,2**	**SEM**	**0,02**
**Pregnancy Mouse**	**YFP+ K14+**	**% K14+ in YFP+**	**YFP+ K8+**	**% K8+ in YFP+**	**K14+**	**% YFP+ in K14+**	**K8+**	**% YFP+ in K8+**
**#1**	1560	100	0	0	1606	97,1	8139	0
**#2**	1624	100	0	0	1652	98,6	7781	0
**#3**	219	99,5	1	0,5	222	98,7	613	0,2
	**Mean**	**99,9**	**Mean**	**0,15**	**Mean**	**98,1**	**Mean**	**0,06**
	**SEM**	**0,15**	**SEM**	**0,15**	**SEM**	**0,5**	**SEM**	**0,06**
**Lactation Mouse**	**YFP+ K14+**	**% K14+ in YFP+**	**YFP+ K8+**	**% K8+ in YFP+**	**K14+**	**% YFP+ in K14+**	**K8+**	**% YFP+ in K8+**
**#1**	1034	98,3	18	1,7	1034	100	11702	0,2
**#2**	904	98,8	11	1,2	904	100	9495	0,1
**#3**	97	96	4	4	97	100	813	0,5
**#4**	165	100	0	0	165	100	1375	0
	**Mean**	**98,3**	**Mean**	**1,7**	**Mean**	**100**	**Mean**	**0,2**
	**SEM**	**0,83**	**SEM**	**0,83**	**SEM**	**0**	**SEM**	**0,1**

Wuidart et al, Supplemental Table 5

Supporting data for Figure 7E – K14rtTA/TetO-Cre/Rosa-YFP TIPS						
**No Chase Mouse**	**YFP+ K14+**	**K14+**	**% YFP+ in K14+**	**YFP+ K8+**	**K8+**	**% YFP+ in K8+**
**#1**	2634	2871	91,7	22	7465	0,3
**#2**	2249	2615	86	16	6799	0,2
**#3**	2917	3280	88,9	25	8528	0,3
**#4**	2342	2602	90	18	6765	0,3
		**Mean**	**89,2**		**Mean**	**0,3**
		**SEM**	**1,2**		**SEM**	**0,01**
**Chase 2W Mouse**	**YFP+ K14+**	**K14+**	**% YFP+ in K14+**	**YFP+ K8+**	**K8+**	**% YFP+ in K8+**
**#1**	1856	2167	85,6	1564	11919	13,1
**#2**	991	1555	63,7	2329	8553	27,2
**#3**	1991	2954	67,4	2652	16247	16,3
		**Mean**	**72,3**		**Mean**	**18,9**
		**SEM**	**6,8**		**SEM**	**4,3**

Supporting data for Figure 7K – K8rtTA/TetO-Cre/Rosa-tdTomato - TIPS						
**No Chase Mouse**	**TOM+ in K14+ cells**	**K14+**	**% TOM+ in K14+**	**TOM+ in K8+ cells**	**K8+**	**% TOM+ in K8+**
**#1**	8	2345	0,3	4738	6097	77,7
**#2**	14	2207	0,6	5122	5738	89,3
**#3**	52	2861	1,8	6301	7439	84,7
		**Mean**	**0,9**		**Mean**	**83,9**
		**SEM**	**0,5**		**SEM**	**3,4**
**Chase 2W Mouse**	**TOM+ K14+**	**K14+**	**% TOM+ in K14+**	**TOM+ K8+**	**K8+**	**% TOM+ in K8+**
**#1**	53	3623	1,5	9311	19927	46,7
**#2**	48	2067	2,3	6892	11369	60,6
**#3**	77	1389	5,5	5380	7640	70,4
		**Mean**	**3,1**		**Mean**	**59,3**
		**SEM**	**1,2**		**SEM**	**6,9**
**Chase 4W Mouse**	**TOM+ in K14+ cells**	**K14+**	**% TOM+ in K14+**	**TOM+ in K8+ cells**	**K8+**	**% TOM+ in K8+**
**#1**	37	1640	2,3	5901	11275	52,3
**#2**	31	1656	1,9	5205	10378	50,2
		**Mean**	**2,1**		**Mean**	**51,2**
		**SEM**	**0,2**		**SEM**	**1,1**

Supporting data for Supplemental Figure 1C – Lgr5-EGFP-IRES-CreER^{T2}/Rosa-tdTomato – 1,5mg TAM - Adulthood					
**48h Mouse**	**K14+ TOM+**	**% K14+ in TOM+**	**K8+ TOM+**	**% K8+ in TOM+**	**Total TOM+ cells**
**#1**	435	78,9	154	26,1	589
**#2**	743	95,1	38	4,9	781
**#3**	527	95,6	24	4,4	551
**#4**	827	91,3	79	8,7	906
	**Mean**	**89**	**Mean**	**11**	

Supporting data for Supplementary Figure 1D – Lgr5-EGFP-IRES-CreER^{T2}/Rosa-tdTomato - Adulthood						
**1,5mg 1w**	**K14+ TOM+**	**K14+**	**% K14+ in TOM+**	**K8+ TOM+**	**K8+**	**% K8+ in TOM+**
**#1**	703	31087	2,26	206	93179	0,22
**#2**	265	23843	1,11	356	71588	0,5
**#3**	852	54272	1,57	483	162664	0,3
		**Mean**	**1,65**		**Mean**	**0,34**
		**SEM**	**0,33**		**SEM**	**0,08**
**15mg 1w**	**K14+ TOM+**	**K14+**	**% K14+ in TOM+**	**K8+ TOM+**	**K8+**	**% K8+ in TOM+**
**#1**	567	35500	1,6	0	106400	0
**#2**	376	9230	4,07	13	27664	0,05
**#3**	2470	86620	2,85	798	259616	0,31
**#4**	181	14200	1,27	0	42560	0
		**Mean**	**2,45**		**Mean**	**0,09**
		**SEM**	**0,64**		**SEM**	**0,07**

Supporting data for Supplementary Figure 2B – K14CreER^{T2}/Rosa -Confetti – 1,5mg TAM + BrdU – Puberty				
**3days Mouse**	**K14+ CFT+ BrdU+**	**K14+ CFT+ BrdU-**	**% BrdU+ in K14+ CFT+**	**% BrdU- in K14+ CFT +**
**#1**	6	11	**35,3**	**64,7**
**#2**	14	40	**25,9**	**74,1**
**#3**	29	173	**14,4**	**85,6**
		**Mean**	**25,2**	**74,8**
		**SEM**	**6,1**	**6,1**
**3days Mouse**	**K8+ CFT+ BrdU+**	**K8+ CFT+ BrdU-**	**% BrdU+ in K8+ CFT+**	**% BrdU- in K8+ CFT +**
**#1**	2	13	**13,3**	**86,7**
**#2**	19	55	**25,7**	**74,3**
**#3**	16	48	**25**	**75**
		**Mean**	**21,3**	**78,7**
		**SEM**	**4**	**4**

Wuidart et al, Supplemental Table 8

Supporting data for Supplementary Figure 3B – K14CreER^{T2}/Rosa -Confetti – 0,05mg TAM – Puberty						
**3days Mouse**	**K14+ CFT+**	**K14+**	**% K14+ in CFT+**	**K8+ CFT +**	**K8+**	**% K8+ in CFT +**
**#1**	202	8268	3,04	3	24803	0,02
**#2**	123	5394	3,26	3	17422	0,05
		**Mean**	**3,15**		**Mean**	**0,03**
		**SEM**	**0,11**		**SEM**	**0,02**

Supporting data for Supplementary Figure 3H– K14CreER^{T2}/Rosa-Confetti – 0,25mg TAM – Adulthood						
**1W Mouse**	**K14+ CFT+**	**K14+**	**% K14+ in CFT+**	**K8+ CFT +**	**K8+**	**% K8+ in CFT +**
**#1**	120	6566	2,19	8	19698	0,05
**#2**	94	4929	2,02	2	14787	0,01
		**Mean**	**2,1**		**Mean**	**0,03**
		**SEM**	**0,08**		**SEM**	**0,02**

Wuidart et al, Supplemental Table 9

Supporting data for Supplemental Figure 4C – K14rtTA/TetO-Cre/Rosa-YFP<br>TOTAL EPITHELIUM						
**No Chase Mouse**	**YFP+ K14+**	**K14+**	**% YFP+ in K14+**	**YFP+ K8+**	**K8+**	**% YFP+ in K8+**
**#1**	4533	4896	92,6	34	12730	0,3
**#2**	4809	5407	88,9	36	14058	0,3
**#3**	4177	4713	88,6	30	12254	0,2
**#4**	5021	5687	88,3	23	14786	0,2
		**Mean**	**89,6**		**Mean**	**0,2**
		**SEM**	**1**		**SEM**	**0,03**
**Chase 2W Mouse**	**YFP+ K14+**	**K14+**	**% YFP+ in K14+**	**YFP+ K8+**	**K8+**	**% YFP+ in K8+**
**#1**	4550	5390	84,4	1958	29645	6,6
**#2**	2475	3906	63,4	2545	21483	11,9
**#3**	2349	3423	68,6	2683	18827	14,3
		**Mean**	**72,1**		**Mean**	**10,9**
		**SEM**	**6,3**		**SEM**	**2,3**

Supporting data for Supplemental Figure 4D – K14rtTA/TetO-Cre/Rosa-YFP DUCTS						
**No Chase Mouse**	**YFP+ K14+**	**K14+**	**% YFP+ in K14+**	**YFP+ K8+**	**K8+**	**% YFP+ in K8+**
**#1**	1899	2025	93,8	12	5265	0,2
**#2**	2560	2792	91,7	20	7259	0,3
**#3**	1260	1433	87,9	5	3726	0,1
**#4**	2679	3085	86,8	5	8021	0,1
		**Mean**	**90,1**		**Mean**	**0,2**
		**SEM**	**1,6**		**SEM**	**0,05**
**Chase 2W Mouse**	**YFP+ K14+**	**K14+**	**% YFP+ in K14+**	**YFP+ K8+**	**K8+**	**% YFP+ in K8+**
**#1**	2694	3223	83,6	394	17727	2,2
**#2**	1484	2351	63,1	216	12931	1,7
**#3**	358	469	76,3	31	2580	1,2
		**Mean**	**74,3**		**Mean**	**1,7**
		**SEM**	**6**		**SEM**	**0,3**

Supporting data for Supplemental Figure 4H – K8rtTA/TetO-Cre/Rosa-tdTomato<br><br>TOTAL EPITHELIUM						
**No Chase Mouse**	**TOM+ K14+**	**K14+**	**% TOM+ in K14+**	**TOM+ K8+**	**K8+**	**% TOM+ in K8+**
**#1**	13	4901	0,3	9158	14703	75,6
**#2**	65	5928	1,1	14545	17784	95,1
**#3**	91	5208	1,7	11155	15624	84,7
		**Mean**	**1**		**Mean**	**85,2**
		**SEM**	**0,4**		**SEM**	**5,6**
**Chase 2W Mouse**	**TOM+ K14+**	**K14+**	**% TOM+ in K14+**	**TOM+ K8+**	**K8+**	**% TOM+ in K8+**
**#1**	119	8631	1,4	27502	47471	57,9
**#2**	86	4735	1,8	17412	26043	66,9
**#3**	105	1873	5,6	7655	10302	74,3
		**Mean**	**2,9**		**Mean**	**66,4**
		**SEM**	**1,3**		**SEM**	**4,7**
**Chase 4W Mouse**	**TOM+ K14+**	**K14+**	**% TOM+ in K14+**	**TOM+ K8+**	**K8+**	**% TOM+ in K8+**
**#1**	54	3690	1,5	16925	25652	66
**#2**	40	2886	1,4	12541	19721	65,1
		**Mean**	**1,4**		**Mean**	**65,6**
		**SEM**	**0,03**		**SEM**	**0,5**

Supporting data for Supplemental Figure 4I – K8rtTA/TetO-Cre/Rosa-tdTomato - DUCTS						
**No Chase Mouse**	**TOM+ in K14+ cells**	**K14+**	**% TOM+ in K14+**	**TOM+ in K8+ cells**	**K8+**	**% TOM+ in K8+**
**#1**	5	2556	0,2	4420	6646	66,5
**#2**	29	3721	0,8	9423	9675	97,4
**#3**	38	2347	1,6	4854	6102	79,5
		**Mean**	**0,9**		**Mean**	**81,1**
		**SEM**	**0,4**		**SEM**	**9,0**
**Chase 2W Mouse**	**TOM+ K14+**	**K14+**	**% TOM+ in K14+**	**TOM+ K8+**	**K8+**	**% TOM+ in K8+**
**#1**	66	5008	1,3	18191	27544	66,0
**#2**	38	2668	1,4	10520	14674	71,7
**#3**	28	484	5,8	2275	2662	85,5
		**Mean**	**2,8**		**Mean**	**74,4**
		**SEM**	**1,5**		**SEM**	**5,9**
**Chase 4W Mouse**	**TOM+ in K14+ cells**	**K14+**	**% TOM+ in K14+**	**TOM+ in K8+ cells**	**K8+**	**% TOM+ in K8+**
**#1**	17	2050	0,8	11024	14350	76,8
**#2**	9	1230	0,7	7336	8893	82,5
		**Mean**	**0,8**		**Mean**	**79,7**
		**SEM**	**0,05**		**SEM**	**2,8**
